# *S. mansoni* Bolsters Anti-Viral Immunity in the Murine Respiratory Tract

**DOI:** 10.1371/journal.pone.0112469

**Published:** 2014-11-14

**Authors:** Sebastian Scheer, Christine Krempl, Carsten Kallfass, Stefanie Frey, Thilo Jakob, Gabriel Mouahid, Hélène Moné, Annette Schmitt-Gräff, Peter Staeheli, Marinus C. Lamers

**Affiliations:** 1 Max Planck Institute of Immunobiology and Epigenetics, Freiburg, Germany; 2 International Max Planck Research School of Molecular and Cellular Biology, Freiburg, Germany; 3 University of Freiburg, Freiburg, Germany; 4 Institute of Virology and Immunology, University of Wuerzburg, Wuerzburg, Germany; 5 Institute for Virology, Department of Medical Microbiology and Hygiene, University Medical Center Freiburg, Freiburg, Germany; 6 Allergy Research Group, Department of Dermatology, University Medical Center Freiburg, Freiburg, Germany; 7 Univ. Perpignan Via Domitia, Ecologie et Evolution des Interactions, UMR 5244, F-66860, Perpignan, France; 8 CNRS, Ecologie et Evolution des Interactions, UMR 5244, F-66860, Perpignan, France; 9 Institute of Pathology, University of Freiburg, Freiburg, Germany; Indiana University, United States of America

## Abstract

The human intestinal parasite *Schistosoma mansoni* causes a chronic disease, schistosomiasis or bilharzia. According to the current literature, the parasite induces vigorous immune responses that are controlled by Th2 helper cells at the expense of Th1 helper cells. The latter cell type is, however, indispensable for anti-viral immune responses. Remarkably, there is no reliable literature among 230 million patients worldwide describing defective anti-viral immune responses in the upper respiratory tract, for instance against influenza A virus or against respiratory syncitial virus (RSV). We therefore re-examined the immune response to a human isolate of *S. mansoni* and challenged mice in the chronic phase of schistosomiasis with influenza A virus, or with pneumonia virus of mice (PVM), a mouse virus to model RSV infections. We found that mice with chronic schistosomiasis had significant, systemic immune responses induced by Th1, Th2, and Th17 helper cells. High serum levels of TNF-*α*, IFN-*γ*, IL-5, IL-13, IL-2, IL-17, and GM-CSF were found after mating and oviposition. The lungs of diseased mice showed low-grade inflammation, with goblet cell hyperplasia and excessive mucus secretion, which was alleviated by treatment with an anti-TNF-*α* agent (Etanercept). Mice with chronic schistosomiasis were to a relative, but significant extent protected from a secondary viral respiratory challenge. The protection correlated with the onset of oviposition and TNF-*α*-mediated goblet cell hyperplasia and mucus secretion, suggesting that these mechanisms are involved in enhanced immune protection to respiratory viruses during chronic murine schistosomiasis. Indeed, also in a model of allergic airway inflammation mice were protected from a viral respiratory challenge with PVM.

## Introduction

Immunity to intracellular pathogens like viruses and mycobacteria is ensured by type I immune responses. These responses are dependent on and characterised by IFN-*γ* [1]. In contrast, infestation with helminths, including that by the parasitic trematode worms of the genus *Schistosoma*, typically elicits host type-II immune responses that are characterised by a massive production of the cytokines IL-4, IL-5 and IL-13 [2], [3]. These cytokines are thought to inhibit the induction of type I immune responses [1], [3], [4]. Helminthic infections are moreover often brought in context with a generalised immune regulation or suppression. Improved hygienic conditions, in which helminth infections have become rare, are now often implied in diseases that are caused by unbalanced immune system activity, like autoimmune diseases and allergy. This is subsumised in the hygiene hypothesis [5]. In this passed-on framework of mutually exclusive immune response types and generalised immunosuppression in worm infestation, anti-viral and anti-mycobacterial responses should be severely hampered in areas with a high spread of *Schistosoma* parasites. However, an unambiguous disease pattern was not found [6], [7].

Schistosomiasis or bilharzia is a chronic and, when untreated, finally lethal parasitic disease; approximately 230 million people worldwide require treatment yearly [8]. Murine schistosomiasis caused by *S. mansoni* resembles human schistosomiasis in many, but not all respects [9]. The pathogenesis involves a passage of the helminth through several organ systems and elicits several types of immune defence mechanisms. An initial Th1-biased host immune response is induced by the invasion of the infectious cercariae. The immature worms (schistosomula) travel through the lungs and liver to their final destination in the portal veins, where they mate. Around 6 weeks after entry, egg deposition by the worm pairs starts. The Th1-biased immune response converts into a Th2-biased response, and is accompanied by a regulatory (suppressive) response with the production of the prototypical cytokine IL-10 [10]. Eggs must travel through the walls of vessel and gut to reach the gut lumen and to leave the host to continue their life cycle. The passage through the gut wall affects its integrity, requiring repair mechanisms and an increased anti-bacterial response, and is accompanied by extensive inflammation. Repair mechanisms are induced by IL-4 and IL-13 [11], whereas defence to extracellular bacteria is dependent on Th17 responses with the signature cytokine IL-17 [12]. Not all eggs make it to the gut lumen. Many are flushed into the liver or are stuck in the gut wall, where they are then encapsulated by a fibrotic granulomatous response.

Coinfection studies in animal models which could have shed light on the anomalous comorbidity situation were also not conclusive and often difficult to interpret: hepatotropic viruses like HCV and LCMV target an organ, which is stressed by the granulomatous reaction and which is extremely sensitive to cytopathologic effects of TNF-*α* and IL-12 [13]–[15].

The lack of expected comorbidities and the ambiguous animal coinfection studies are puzzling in the light of the predominant immunological dogmas. More study is required, with emphasis on systemic effects. The observations also raise the question, whether, from an evolutionary point of view, the parasitic relationship does not only have an unfavourable, but also a certain beneficial effect on the host [16]. Further, recent evidence points to the (gut) microbiome as a modulator of immune responses, also in distant locations like the airways, to respiratory viruses [17], [18].

In the present study we infected mice, without antibiotic treatment, with a human isolate of *S. mansoni* from Oman. We tested the immune response in the chronic phase of the disease, to viruses with tropism for tissues that are not directly targeted in schistosomiasis. Hereto we have chosen the respiratory tract viruses pneumonia virus of mice (PVM) [19] and influenza A [20]. We show that a primary, chronic infection with the Omani isolate induces strong, coexistent, and systemic cytokine secretion of the Th1, Th2, and Th17 type. In particular, high serum levels of TNF-*α* were found that induced a low-grade inflammatory response and a marked hyperplasia of mucus-producing cells in the lung. Under these conditions, mice were relatively well protected from a secondary infectious challenge with the pneumotropic viruses influenza A and PVM. This suggests that chronic schistosomiasis can provide a beneficial effect on the host by reducing the susceptibility to respiratory viral infections.

## Materials and Methods

### Ethics Statement

Animal experimentation was approved by the local animal welfare authority (Regierungspräsidium Freiburg, Abt. 3, Referat 35; reference: AZ.: 35/9185.81/G-08/104), based on local law and animal ethics regulations (Tierschutzgesetz: http://www.gesetze-im-internet.de/bundesrecht/tierschg/gesamt.pdf). The health status of the mice was scored with some modifications according to Morton and Griffiths [21]. Criteria used for termination were: appearance (grooming, starey coat, posture, dehydration, crusty eyes or nose); body weight (slight (<5%), moderate (5–10%), moderate with decreased appetite (10–20%), severe (>20%); clinical symptoms (breathing rate, heart rate, body temperature, diarhoea, anaemia); handling (alertness, agressiveness, irritability); and behaviour (self-mutilation). Animal condition was monitored on a daily basis, if necessary, at shorter intervals. Mice were sacrificed by asphyxia in carbondioxide.

### Mice

Mice were bred and kept in the SPF animal facility of the Max-Planck-Institute of Immunobiology and Epigenetics or of the Virology Department of the University of Freiburg, without antibiotic treatment. B6.A2G-*Mx1* mice carrying functional Mx1 alleles were previously described [20].

### Infections

#### *S. mansoni*

An Omani human isolate [22] was maintained using *Biomphalaria* species as intermediate hosts. Mice were anaesthetized with Ketamine/Xylazine (0.1 mg resp. 0.5 mg/mouse) and exposed to 80–90 cercariae via the shaved belly method [23]. Infection efficiency was ∼75% (number of cercariae left after 1 h of exposure/number of input cercariae). Mice were infected at 10–11 weeks of age.

#### Influenza

Mice were anaesthetized with Ketamine/Xylazine and infected intranasally (i.n.) with either influenza A strain hvPR8 or strain PR8. For influenza A strain hvPR8 (a highly virulent variant of A/PR/8/34 [20]) Mx1-competent mice received 60 or 200 pfu in 50 µl MEM. Mx1-competent control mice received 5×10^5^ U hybrid human IFN-*α*B/D one day before virus challenge. For influenza A strain PR8 C57BL/6J mice (Mx1-deficient) received 2000 pfu i.n. in 50 µl MEM. Challenge was at 10–12 weeks after primary infection with *S. mansoni*. Control mice were left untreated until the infection with influenza virus.

#### PVM

Mice were anaesthetized with isoflurane (Baxter) and infected i.n. with either 200 or 2000 pfu of PVM clone 15 (ATCC) in 80 µl MEM medium. Virus titers were determined as described previously [24]. Viral challenge was at 12 weeks after primary infection with *S. mansoni*, unless stated otherwise. Control mice were infected at 12–15 weeks of age.

After infection, mice were observed daily and weighed at indicated time points at the same time of day.

### Treatment with Etanercept

Mice received Etanercept (a kind gift of Pfizer Germany) at 4 mg/kg body weight intraperitoneally at the indicated time points.

### Immunisation with OVA

Mice were immunised i.p. with 20 µg chicken egg ovalbumin (OVA; grade V; Sigma-Aldrich) in 200 µl Alum (4 mg/ml in PBS; Imject Alum Adjuvant; ThermoScientific), at day 0, 7 and 14. Two weeks later, the mice were treated 3 times at daily intervals with aerosolised OVA (1% w/v OVA in PBS) for 20 min [25]. 24 h after the last treatment, the mice were either sacrificed and analysed, or infected with 200 pfu/mouse of PVM.

### Broncho-alveolar lavage (BAL)

Lungs were lavaged with 4×800 µl PBS. The BAL fluid (BALF) of the first lavage was used for quantification of cytokines. The cells of the lavages from one mouse were pooled, counted, and used for analysis or *ex vivo* stimulation.

### Organs

After BAL, the right half of the lung was dissected, chopped in pieces (<1 mm^3^) and digested for 1 h with 25 µg/ml Liberase DL and 10 µg/ml DNase I (both Roche) in HBSS medium. The cell suspension was treated with Gey's solution to deplete erythrocytes, washed, filtered through a 100 µm mesh, and used for flow cytometry. The left half of the lung was snap-frozen and stored at −80°C until analysis.

For the assessment of virus entry, lungs were instilled with 800 µl 4% formalin and kept in 4% formalin for 2 h at room temperature. Afterwards, the lungs were washed with PBS, tumbled for 24 h in 30% sucrose at 4°C, washed with PBS, embedded in OCT, and kept at 4°C for another 24 h before storage at −80°C.

### Muc5ac and Muc5b expression

Lungs were snap frozen in liquid nitrogen and stored at −80°C. For RNA extraction, the tissue was extracted with TriReagent (Sigma), and the RNA was immediately reverse-transcribed with SuperScript III (Invitrogen) using random hexamers as primers. Quantitative PCR analysis was performed with a Lightcycler (Roche), 18S RNA was used as internal standard. Primers used were described earlier [26].

### Cytokine analysis

Serum and BALF were stored at −80°C and analysed for IL-2, IL-4, IL-5, IL-6, IL-10, IL-12p70 (IL-12), IL-13, IL-17, GM-CSF, MIP-1*α* (CCL3), TNF-*α*, and IFN-*γ* applying the Pro Cytokine Assay with the Bio-Plex 200 System (both Bio-Rad). IFN-*α* was determined with the Premium ELISA kit (eBioscience). To gauge expression of IFN-*β*, Mx-1-competent mice harbouring a reporter for IFN-*β* [27] were infected with *S. mansoni*. 12 weeks after infection mice were sacrificed, and luciferase activity in lung tissue was determined as described [27].

### Endotoxin determination

Endotoxin levels in the serum of mice was determined by LAL assay (QCL-1000, LONZA).

### Flow cytometry

Single-cell suspensions of organs were treated with Gey's solution where appropriate, and washed with PBS/3% FCS. The cells were preincubated with 20 µg anti-CD16/32 antibody (clone 2.4G2), washed and stained with a combination of fluorochrome-labelled monoclonal antibodies (all steps at 4°C). Antibodies were from BD Biosciences {name [alternative name] clone)} {TCR*β* (H57-597); CD4 (RM4-5 or GK1.5), CD5 ([Ly-1] 53–7.3), CD8 (53–6.7), CD11b (M1/70); CD11c (HL3), CD11c (IM7), CD19 (1D3), Siglec F (E50-2440), NK1.1 (PK136), Ly6G (1A8)}, eBioscience {I-Ab [MHC class II] AF6-120.1), H-2K^b^ [MHC class I] AF6-88.5.5.3), CD3e (eBio500A2 or 145-2C11), CD25 (PC61.5), CD27 (LG.7F9), CD45 (30-F11), CD90.2 (53–2.1), F4/80 (BM8), Ly6C (HK1.4)}, and MD Bioproducts {T1-ST2 (DJ8)}. Samples were processed on a LSR II flow cytometer equipped for 10-parameter acquisition, and analysed with TreeStar FlowJo software (version 9.5).

### Characterization of ILCs and lung interstitial leukocytes

Lungs were digested as above and ILCs were prepared and characterised as described [28] using the lineage markers CD11c, CD3, B220, TCR*β*, CD5, CD27, NK1.1 and CD11b. Lineage^neg^ cells were considered ILCs when they were positive for the markers CD45, CD90, CD25 and T1-ST2. Data shown are from 10, 8, 8, and 14 animals for control mice and from 10, 5, 6, and 4 animals for *S. mansoni*-infected mice at 0, 4, 7, and 10 days post infection with PVM, respectively.

### Histology

Sections of paraffin-embedded lungs were stained with H&E or PAS and analysed for columnar cell hyperplasia, goblet cell hyperplasia, atypica, degenerative changes, epithelial necrosis (for the assessment of involvement of the bronchial mucosa), fibrosis, and inflammation (for the assessment of interstitial disease). Sections were scored blindly by one experienced observer. Scoring was as follows: 0: normal; 1: minor; 2: intermediate; 3: markedly increased pathology. Sections of OCT-embedded lungs were fixed with 75% acetone/25% methanol, rehydrated with PBS, blocked with anti-CD16/32 antibody (clone 2.4G2) and stained with rabbit anti-PVM-antibody specific for the PVM-G protein [24]. They were then washed, blocked with 5% goat serum and counterstained with goat Alexa555-conjugated anti-rabbit IgG (Invitrogen). The sections were embedded with ProLong Gold Antifade containing DAPI (Invitrogen) and analysed on a Zeiss Imager Z1 at 10× magnification (Zeiss, Plan-Apochromat, 0.17) with a Zeiss AxioCam MRc. Data was acquired and processed with Zeiss Axiovision software (release 4.8.2).

### Statistics

Significant differences between two groups were estimated using the unpaired t test with Welch correction for data with Gaussian distribution (passed assumption method of Kolmogorov and Smirnov), and the Mann-Whitney test for data with non-Gaussian distribution. Significant differences between three groups were estimated using two-way ANOVA with Bonferroni post-test. Statistical relevance was indicated in the plots when significant (*p*≤0.05).

## Results

### Systemic cytokine response in C57BL/6 mice infected with *Schistosoma mansoni*

**Figure 1:**
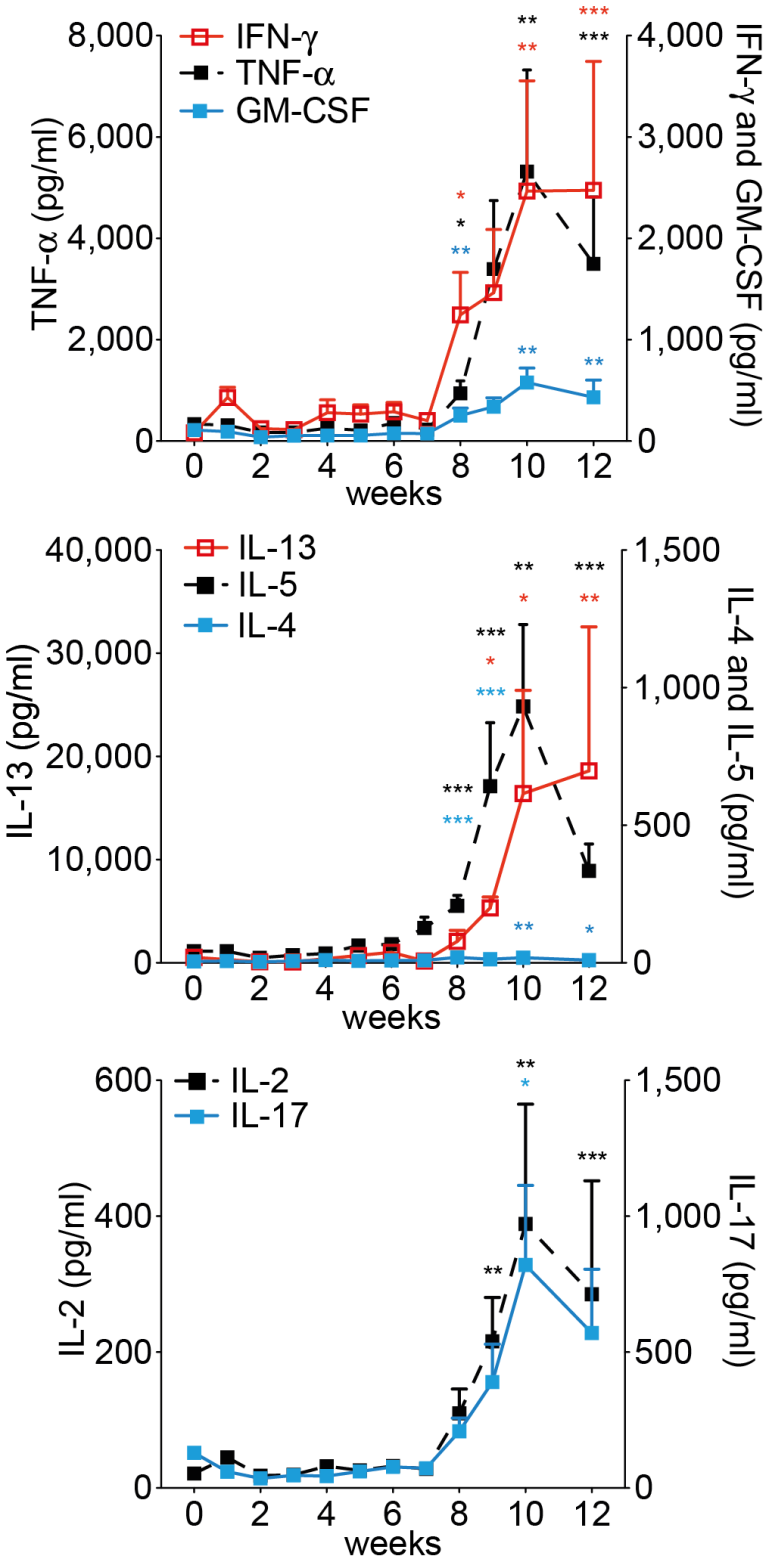
Serum cytokine levels of C57BL/6J mice infected with *S. mansoni*. Cytokine levels were determined at indicated time points after infection. The data points represent means from 4–10 mice. Error bars represent SEM. Significant differences between each time point and control mice (day 0) are marked: *, *p*≤0.05; **, *p*≤0.01; ***, *p*≤0.001.

In our studies we used a recent human isolate of *S. mansoni*, which originated from Oman [22], [29]. We first established the optimal number of cercariae that caused chronic schistosomiasis in C57Bl/6 mice (Fig. S1). 70–75 cercariae per mouse, infected at 9–10 weeks of age, was considered optimal. Herewith a moderate, chronic course of the disease was established that resembled Henderson's moderate splenomegaly syndrome (MSS) of mouse schistosomiasis [30], and that allowed the study of secondary infections in the chronic phase of the disease. Survival at 16 weeks of age was more than 95% in a large group of animals that were infected at different time points during this study. Serum cytokine profiles were also established. As expected, a slight increase in IFN-*γ* levels was seen immediately after infection, indicative of Th1-type immune responsiveness. Significant Th2-type responsiveness was seen from week 7 of infection onwards, after the onset of oviposition, with IL-4 and IL-5 rising ahead of IL-13. Also, IL-17 and IL-2 levels rose significantly after week 7. We found very high systemic levels of the proinflammatory cytokines IFN-*γ*, TNF-*α* and GM-CSF after week 7, which challenges the idea of mutually exclusive immune responses (Fig. 1). IL-10 showed small peaks of activity at week 1, 4, and 8, which most likely reflected a reaction to inflammatory processes after skin and lung passage of the schistosomula, and egg passage into the gut [31].

These findings were not dependent on the Th1-prone mouse strain C57BL/6J; in Th2-prone BALB/c mice we obtained similar data, with the exception of GM-CSF, which did not increase (Fig. S2).

We also analysed the ability of T cells from spleen and mesenteric and pulmonary (mediastinal) lymph nodes to secrete cytokines after polyclonal stimulation. The T cells produced high levels of the Th1-type cytokines *in vitro*, also later in the infection (Table S1). However, the increase of Th1 cytokines was less profound than those of Th2 cytokines, in agreement with previous reports [3].

The high levels of serum IFN-*γ*, but not those for TNF-*α* during an infection with *S. mansoni* is dependent on ligands for TLR2 and/or TLR4, because in mice deficient for both TLRs serum IFN-*γ* was low throughout the infection (Fig. S3). In IL-12R*β*2-deficient mice neither a systemic IFN-*γ*, nor a TNF-*α* response was found (Fig. S3). We could not detect increased systemic levels of endotoxin in mice that were infected with *S. mansoni* (Fig. S4).

We conclude that the immune response in C57BL/6 mice to a chronic infection with *S. mansoni* is not polarised systemically. High levels of the signature Th1 cytokine IFN-*γ* are dependent on TLR2 or 4 ligands, both TNF-*α* and IFN-*γ* are dependent on IL12R*β*2.

### Influence of chronic schistosomiasis on the lung environment

**Figure 2:**
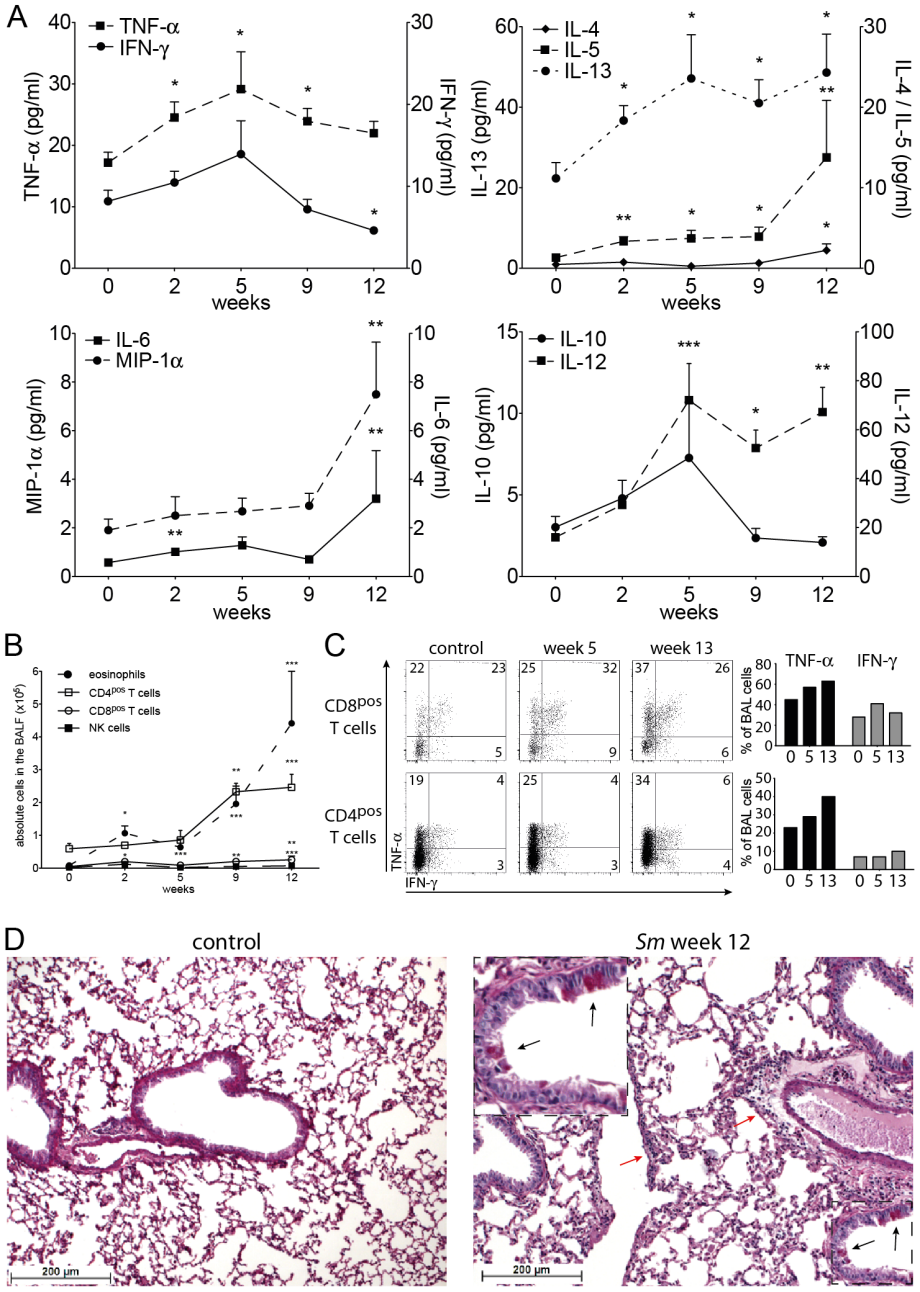
Immuno- and histopathology of lungs of C57BL/6J mice with schistosomiasis. **A**, BALF cytokine levels at indicated time points after infection with *S. mansoni*. Data was cumulatively acquired from 6–9 mice in 3–4 experiments; **B**, Number of different cell types in the BALF of mice with schistosomiasis, at indicated times after infection. Data points represent means of 13 (week 0 of infection), 4 (week 2), 7 (week 5), 9 (week 9) and 14 mice (week 12) in 2–5 individual experiments. **C**, Potential of CD4^pos^ and CD8^pos^ BALF cells to produce IFN-*γ* and TNF-*α* 4 hrs after stimulation with PMA/Ionomycin/Brefeldin A, cells taken at the indicated time points after infection, left panels (FACS plots) show intracellular stainings, right plots (bar graphs) show a quantitative rendition of the FACS data; data shown is representative for 3 individual experiments with 3 mice each. **D**, Section of the lung (H&E staining) of a control animal (left) and a mouse 12 wks after infection with *S. mansoni* (right), insert shows goblet cell hyperplasia (black arrows) and light cellular infiltrates (red arrow). Sections are representative for 3 mice each; Error bars represent SEM. Significant differences between each time point and control mice (day 0) are marked: *, *p*≤0.05; **, *p*≤0.01; ***, *p*≤0.001.

The epithelial barrier layer of the lung reacts to environmental stimuli with the production of alarmins like IL-33 and GM-CSF [32]–[34], which attract dendritic cells. These cytokines also stimulate innate lymphoid cells in the lung (ILCs [35], [36]) to produce IL-5 and IL-13, which create an intensely Th2-prone environment, and to produce amphiregulin, which is important for the integrity of the barrier layer [37]. We studied the steady state conditions in the lung during the infection with *S. mansoni* (Fig. 2). Analysis of the broncho-alveolar lavage fluid (BALF) showed a transient increase in the Th1 cytokines IFN-*γ*, TNF-*α* and IL-12 at week 5 post infection. At this time point schistosomula have passed through the lungs and establish in the portal system. Thereafter, IFN-*γ* fell below normal levels, while TNF-*α* and IL-12 remained significantly increased throughout the measurement period. The Th2 cytokines IL-5 and IL-13 increased steadily from week 2 onwards; IL-4 was only increased late in infection. IL-10 was increased around week 5 post infection, while the stress cytokines IL-6 and MIP-1*α* were significantly increased late in infection (Fig. 2A). Thus, also in the BALF we registered concomitant, not mutually exclusive Th1 and Th2 immune responses during the infection with *S. mansoni*.

**Figure 3:**
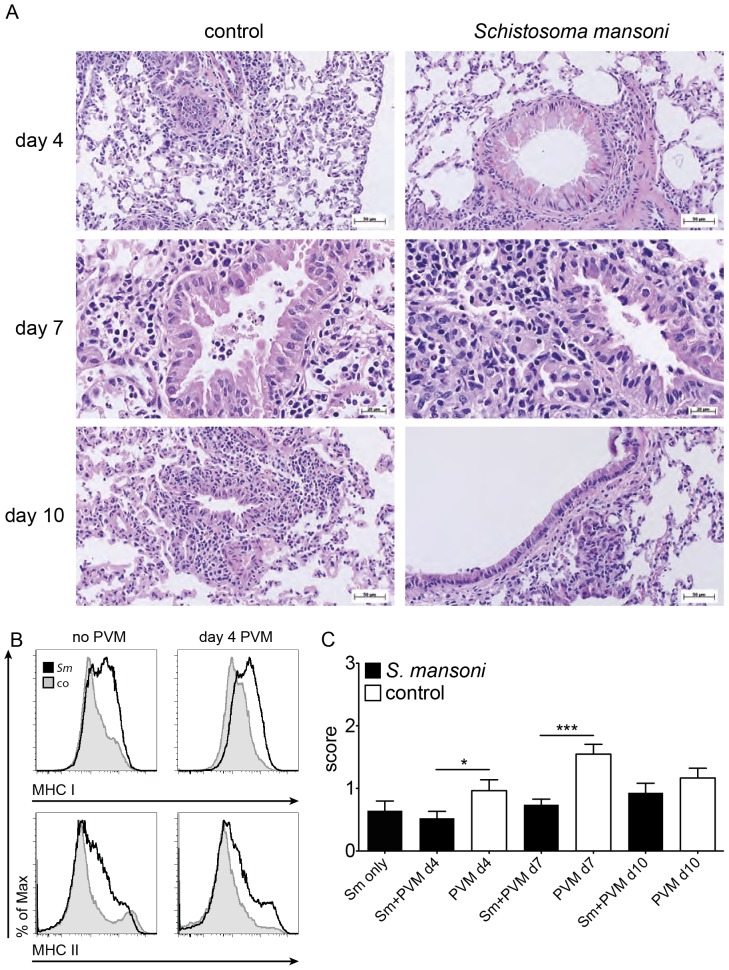
Immuno- and histopathology of lungs of C57BL/6J mice with a coinfection of *S. mansoni* and PVM. **A**, Section of the lung (H&E staining) of control animals (left) and mice 12 wks after infection with *S. mansoni* (right), at indicated time points after infection with PVM (200 pfu/mouse). **B**, FACS profiles of the expression of MHC class I and II antigens on interstitial CD45^pos^ CD11b^pos^ SiglecF^neg^ lung cells of mice with chronic schistosomiasis (12 weeks) and control animals, before and 4 days after challenge with PVM, shaded grey: control mice, black line: mice with schistosomiasis. The data shown are pooled samples from 3 mice and is representative for 9 mice in 3 individual experiments; **C**, Combined histopathological score of mice treated as described in panel A, at the indicated time points after challenge with PVM. Bars represent the mean results from 2–3 mice per group. Scoring: 0: normal; 1: minor; 2: intermediate; 3: markedly increased pathology; Error bars represent SEM. Significant differences are marked: *, *p*≤0.05; **, *p*≤0.01; ***, *p*≤0.001.

We also analysed the cellular composition of the BALF (Fig. 2B). Total cell numbers of eosinophils were increased throughout the infection, with an early peak at week 2 of infection. The number of CD4^pos^ and CD8^pos^ T cells and NK cells were increased late in infection. *In vitro* TNF-*α*secretion by CD4^pos^ and CD8^pos^ T cells was doubled late in infection (Fig. 2C). The epithelial cell layer of the airway showed discrete signs of an increased barrier function with mucus-producing goblet cell hyperplasia, columnar cell hyperplasia, and light perivascular and peribronchial infiltrates (Fig. 2D and Fig. S5A). Furthermore, as signs of increased immunological alertness we saw a marked increase in the expression of MHC class I and II on CD45^pos^ CD11b^pos^ SiglecF^neg^ interstitial lung cells (Fig. 3B, left panels). We did not find an expansion of ILCs, rather we observed a relative contraction of this cell population (Fig. S6A).

We conclude that in murine schistosomiasis a low-grade inflammation is found in the lung with strong signs of an increased barrier function.

### Muc5a expression is increased in mice with chronic schistosomiasis and is inhibited by depletion of TNF-*α*

**Figure 4:**
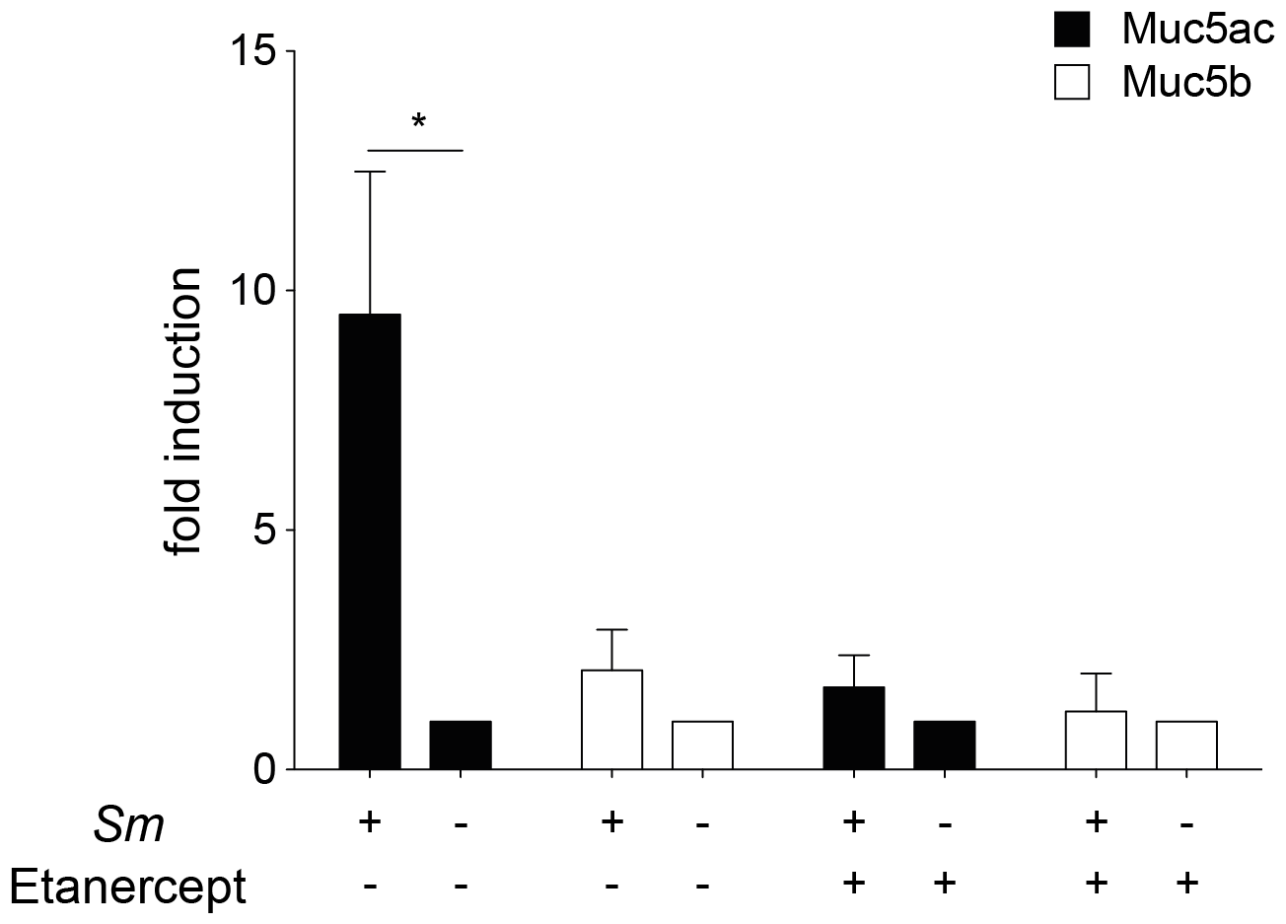
Muc5ac and Muc5b expression in the lungs of mice with chronic schistosomiasis before and after treatment with Etanercept. At 11 week of infection with *S. mansoni*, mice were treated 3 times, every other day, with 4 mg/kg body weight, intraperitoneally. Lungs were taken at day 6 of treatment. Error bars represent SEM. Significant differences between each time point and control mice (day 0) are marked: *, *p*≤0.05.

Goblet cell hyperplasia can be induced, among others, by IL-13 [38] and TNF-*α* [39], [40]. A hallmark of goblet cell hyperplasia is the increased production of the gel-forming mucin Muc5ac [41], [42]. We determined Muc5ac mRNA levels in lungs of mice infected with *S. mansoni* and in control mice. Muc5ac mRNA levels were 10× times higher in infected mice compared to control mice. As expected [42], only a moderate induction of the levels of Muc5b was found in infected animals. We treated mice with soluble TNF-receptor (Etanercept) [43] to explore the role of TNF-*α* in the increased mucus production. After treatment of the mice (starting at week 11 of infection), mRNA for Muc5ac returned to baseline levels (Fig. 4). Thus, we conclude that TNF-*α* is essential for pulmonary goblet cell hyperplasia in chronic schistosomiasis.

### Coinfection of mice with chronic schistosomiasis with influenza A virus

**Figure 5:**
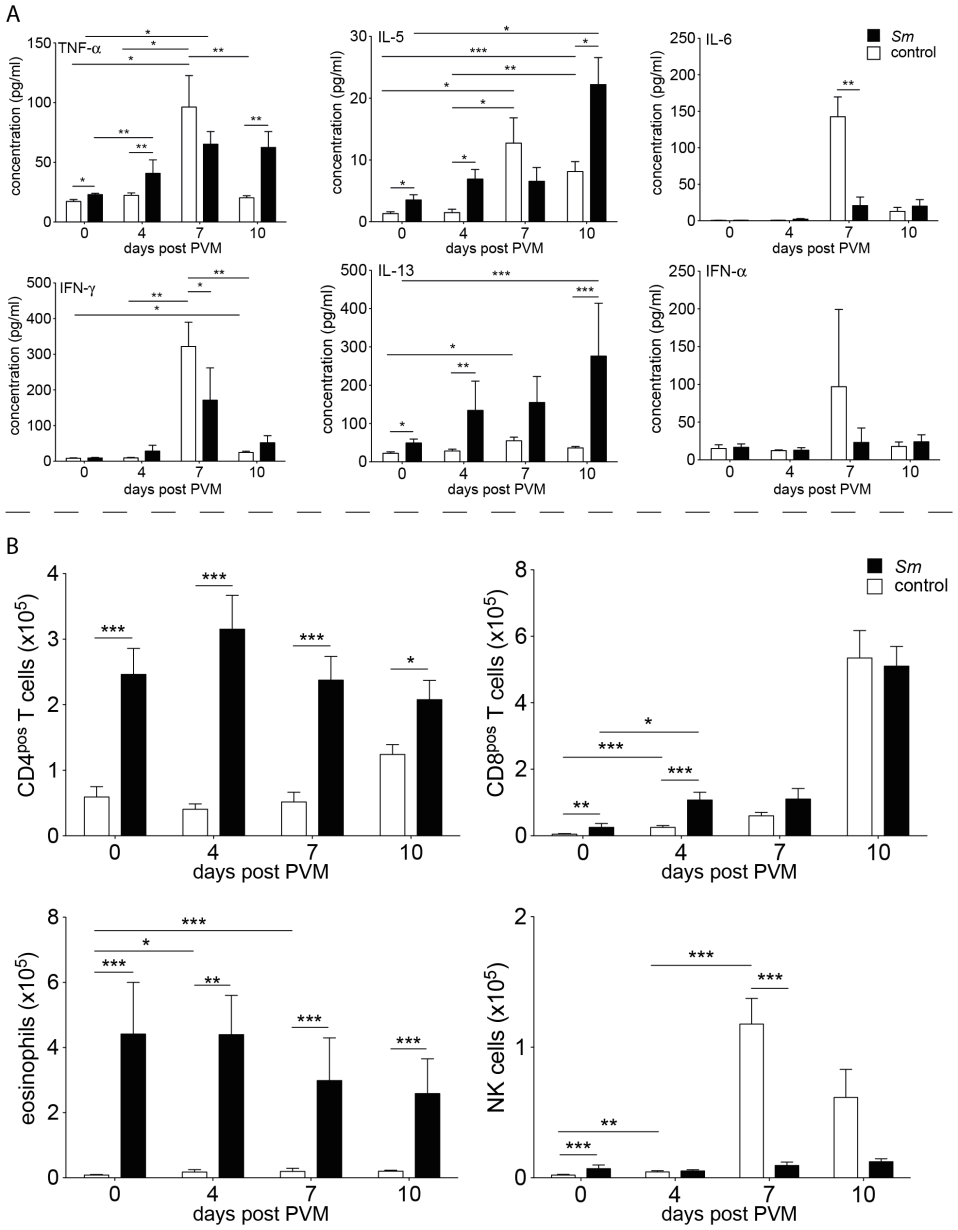
Analysis of BALF and lung tissue of C57BL/6J mice with schistosomiasis and control mice before (day 0) and at indicated time points after infection with PVM (200 pfu/mouse). Filled columns: mice with schistosomiasis, empty columns: control mice. **A**, Cytokine levels. Bars represent data from 7–15 mice in 3–5 individual experiments; **B**, Cell counts for different cell types (CD4^pos^ T cells, CD8^pos^ T cells, eosinophils and NK cells) in the BALF of mice that were infected only with PVM (empty columns), or with *S. mansoni* and challenged 12 wks later with PVM (filled columns) and control mice (empty columns at day 0). Data are from 9–15 mice in 4–5 individual experiments. Error bars represent SEM. Significant differences are marked: *, *p*≤0.05; **, *p*≤0.01; ***, *p*≤0.001.

**Figure 6:**
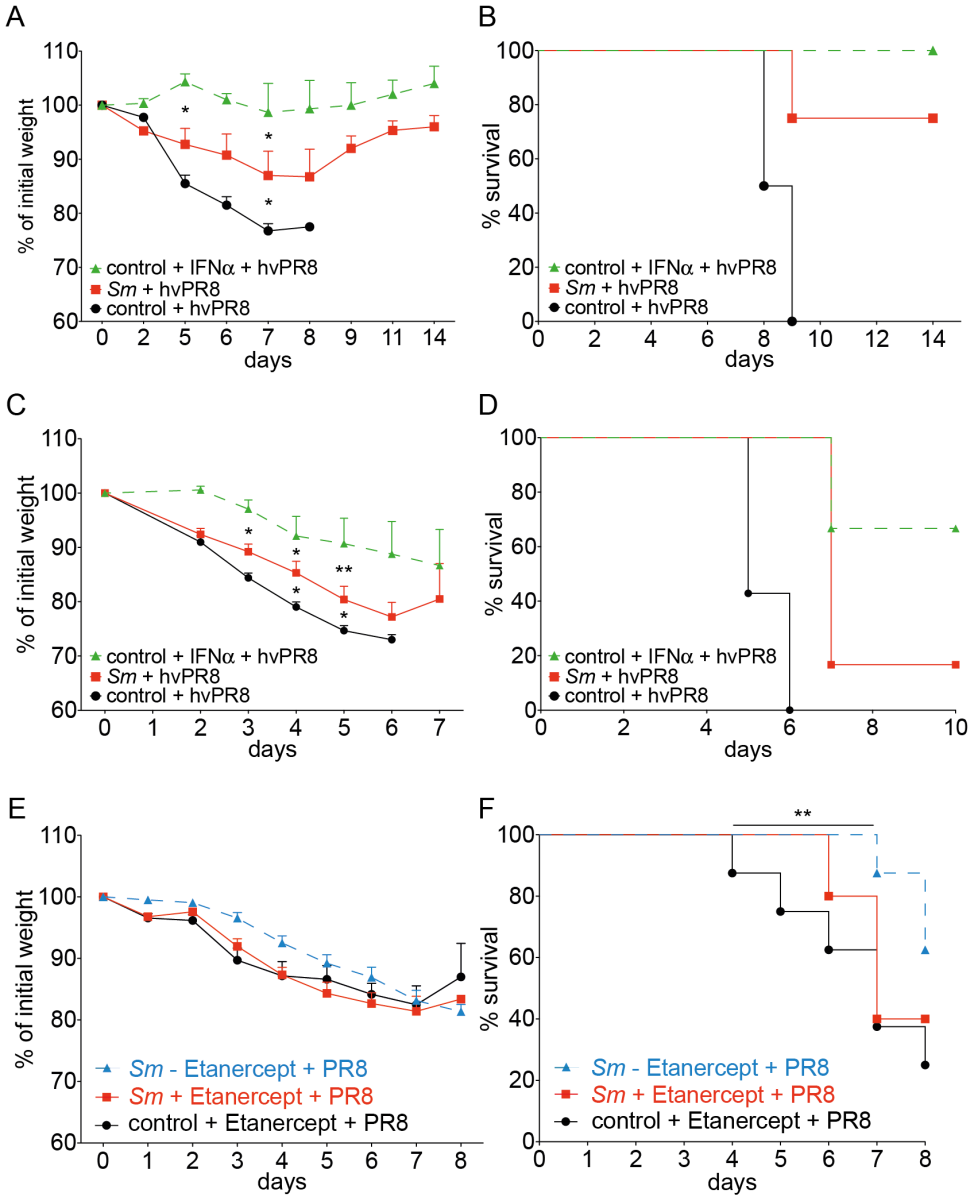
Coinfection experiments with *S. mansoni*-infected C57BL/6J mice and Influenza A virus. **A–D**: Analysis of C57BL/6 mice carrying functional Mx1 alleles (B6.A2G-*Mx1*) after infection with *S. mansoni* and influenza A virus (strain hvPR8). **E–F**: Analysis of wild-type, MX-1-deficient C57BL/6 mice after infection with *S. mansoni* and influenza A virus (strain PR8). **A**, Relative weight loss of mice with chronic schistosomiasis (10 wks; red) and control animals (black) at indicated times after challenge with 60 pfu/mouse of hvPR8. Some control mice received 50.000 U IFN-*α*, given one day before challenge with hvPR8 (green). Data points were cumulatively acquired from 3–4 mice in each group in one experiment. Error bars represent SEM; **B**, Survival curves of mice in Fig. 6A. **C**, Relative weight loss of mice with chronic schistosomiasis (10 wks, red) and control animals (black) at indicated time points after challenge with 200 pfu/mouse of hvPR8. Some control mice received 50.000 U IFN-*α*, given one day before challenge with hvPR8 (green). Data points were cumulatively acquired from 7 mice in each group in one experiment; **D**, Survival curves of mice in Fig. 6C; **E**, Relative weight loss of mice with chronic schistosomiasis (10 wks; red, treated with Etanercept, blue, not treated, black, control animals treated with Etanercept) at indicated time points after challenge with 2000 pfu/mouse. Data points were cumulatively acquired from 7, 5 and 7 mice in each group, respectively, in one experiment; **F**, Survival curves of mice in Fig. 6E; Error bars represent SEM. Significant differences are marked: *, *p*≤0.05; **, *p*≤0.01; ***, *p*≤0.001, nd: below detection limit of 10 pfu.

Increased production of Muc5ac has recently been shown to protect from severe infection with influenza virus [44]. We therefore tested the resistance of mice with chronic schistosomiasis to a secondary infection with influenza. We first used influenza A virus strain hvPR8, which is lethal to normal mice, even if they can express the influenza virus resistance gene Mx1 [45]. However, such mice are protected against hvPR8, if they are pretreated with a single dose of type I IFN [20], because expression of Mx1 is regulated by type I and type III interferons [46]. We found no evidence for the presence of IFN-*α*in the BALF of mice infected with *S. mansoni* (Fig. 5A). Further, IFN-*β*-reporter mice [27] that were chronically infected with *S. mansoni* showed no evidence for enhanced production of IFN-*β* in the lungs. However, IFN-*β* was significantly enhanced in liver and to a lesser extent in the kidney (Fig. S7). We infected Mx1-competent mice in the chronic phase of schistosomiasis with 60 pfu of influenza A strain hvPR8. Mice infected with *S. mansoni,* without pretreatment with IFN, were significantly protected from a challenge with hvPR8, and they survived longer when compared to non-infected (*S. mansoni*-free) controls (Fig. 6A,B). When the challenge dose of influenza virus was increased to 200 pfu per animal, animals with chronic schistosomiasis were still relatively protected and survived longer than control animals (Fig. 6C,D). From these experiments we conclude that infection with *S. mansoni* offers a relative, but significant protection from influenza virus, which is independent of IFN production.

To gauge the role of Muc5ac, we treated Mx1-deficient mice that were infected *S. mansoni* 10 weeks previously with Etanercept or PBS 3 and 1 day before, and also 1 and 3 days after infection with influenza A virus strain PR8 (2000 pfu/mouse). Control mice which were not infected with *S. mansoni* were treated similarly. As expected, control mice were not protected from infection (Fig. 6E,F). However, mice with chronic schistosomiasis were significantly protected with less weight loss and enhanced survival. Treatment with Etanercept abrogated protection, which indicates that the reduced virulence of influenza A virus in mice with chronic schistosomiasis is mediated by a TNF-*α*-induced goblet cell hyperplasia and enhanced Muc5ac synthesis.

### Coinfection of mice with chronic schistosomiasis with pneumonia virus of mice (PVM)

**Figure 7:**
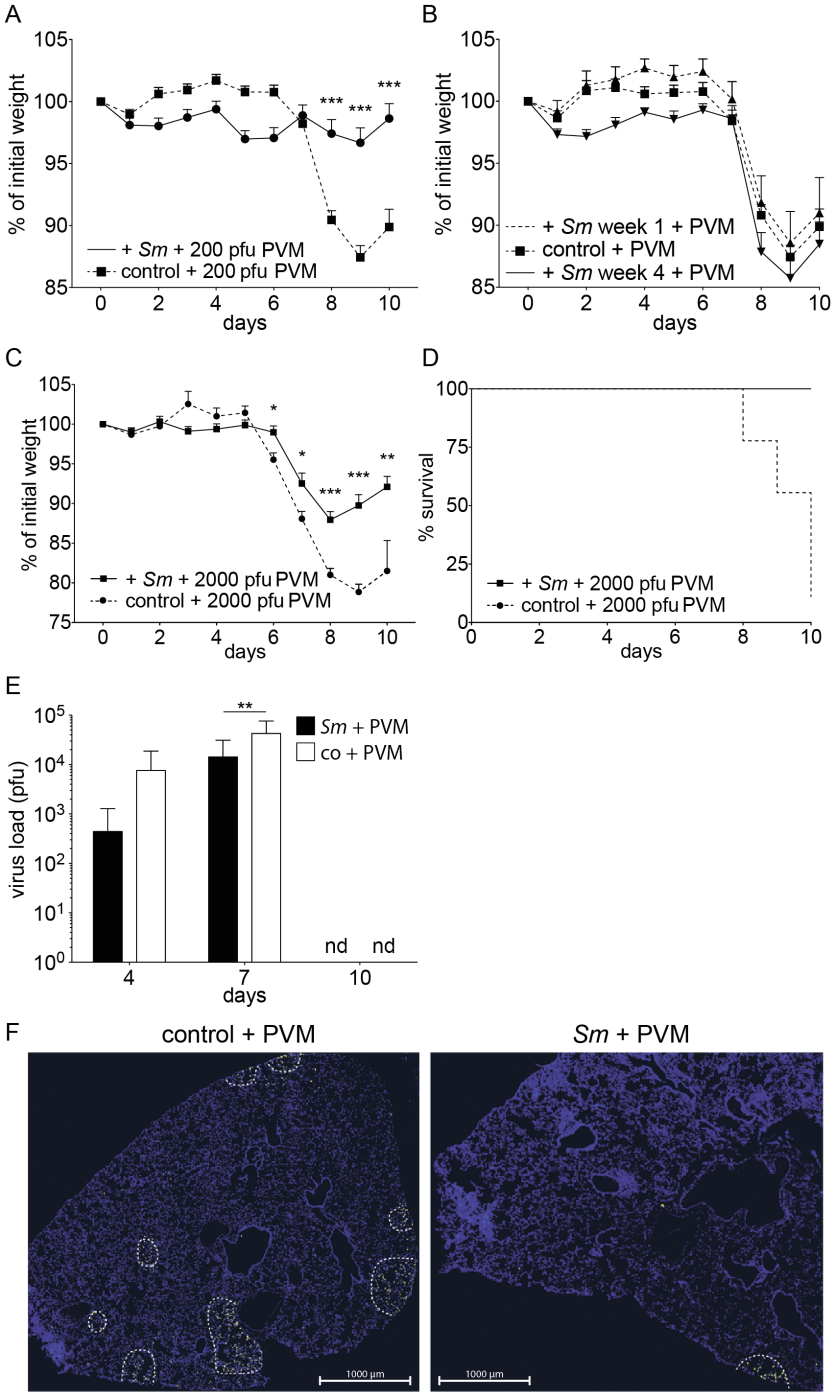
Coinfection experiments with *S. mansoni*-infected C57BL/6J mice and pneumonia virus of mice (PVM). **A**, Relative weight loss of mice with chronic schistosomiasis (12 wks) and control animals at indicated times after a sublethal dose (200 pfu/mouse) of PVM. Data are from 30–38 mice in 7–8 experiments. **B**, Relative weight loss of mice at the indicated time points after challenge with 200 pfu/mouse of PVM, at 1 and 4 weeks after infection with *S. mansoni*. The data are cumulative from 15 (week 1), 6 (week 4), and 52 (control) mice representative of 3, 1 and 12 experiments, respectively; **C**, Relative weight loss of mice with chronic schistosomiasis (12 weeks) and control animals at indicated time points after a lethal dose (2000 pfu/mouse) of PVM. The data are cumulative from 10 mice in 2 individual experiments; **D**, Survival curves of mice in Fig. 7C; **E**, Viral load, expressed as pfu, in the right lobes of the lungs of mice, treated as described in Fig. 7A, at day 4, 7 and 10 after challenge with PVM. Bars represent data from 9–15 mice in 2–4 individual experiments; **F**, Lungs of mice as described in Fig. 7A were taken at day 4, fixed with paraformaldehyde and stained with an antibody directed against the G-protein of PVM. The infected areas are enclosed with dashed lines. Images are representative for 6 sections from 3 individual mice per group; Error bars represent SEM. Significant differences between groups at one time point are marked: *, *p*≤0.05; **, *p*≤0.01; ***, *p*≤0.001, nd: below detection limit.

We also performed coinfection experiments with *S. mansoni*-infected mice and the respiratory virus PVM to validate our findings with a second pneumoviral infection. 12 weeks after infection with *S. mansoni*, mice were challenged intranasally with a sublethal dose (200 pfu/mouse) of PVM. C57BL/6 mice without *S. mansoni* showed, as expected [47], signs of disease and a significant loss of weight 8–9 days after infection, followed by a recovery. In contrast, mice infected with *S. mansoni* 12 weeks before viral challenge did not show additional signs of disease and did not lose weight (Fig. 7A). Viral challenge shortly after infection with *S. mansoni* did not cause protection (Fig. 7B), in fact, reproducible protection was only found after week 8 of infection (data not shown). Whereas viral challenge with 2000 pfu/mouse was lethal in control mice, mice with chronic (12 weeks) schistosomiasis survived this type of challenge, but they did show signs of disease (Fig. 7C,D). Sublethally infected T cell-deficient mice fail to clear the virus, but they are able to exert some virus control, and the virus load can stay constant over weeks without extensive disease or weight loss [47]. The reported dominant Th2 response and immune regulatory activity in the chronic phase of schistosomiasis prompted the question, whether mice with schistosomiasis controlled or cleared the virus. Fig. 7E shows that mice with schistosomiasis carried a significantly reduced (*p*≤0.01) viral burden at day 7 post PVM infection and were able to clear the infection. Immunohistochemical staining of the lungs corroborated this finding (Fig. 7F).

Before viral challenge we found increased levels of TNF-*α*, IL-5, and IL-13 in the BALF of mice with chronic schistosomiasis compared to healthy controls (Fig. 2A, week 12 and Fig. 5A, day 0). At day 4 after viral challenge we found significantly higher levels of TNF-*α*, IL-5 and IL-13 in mice with chronic schistosomiasis, whereas at day 7 control animals had significantly higher levels of IFN-*γ* and IL-6, but lower levels of IL-13. At day 10 after challenge, the titers of TNF-*α*, IL-5 and IL-13 remained high in *Schistosoma*-infected mice and were significantly increased compared to control animals, all of which showed falling titers for all measured cytokines. As expected [48], IFN-*α*, a type I interferon, was markedly increased at day 7 after viral challenge in the BALF of control animals, but was not increased in *Schistosoma*-infected mice (Fig. 5A).

CD4^pos^ T cells, CD8^pos^ T cells, NK cells, and eosinophils were present in significantly higher numbers at all times after viral challenge in the BALF of *Schistosoma*-infected mice when compared to control animals, with the exception of day 7 and 10, where we found highly increased numbers of NK cells in control mice (Fig. 5B). At day 4 after viral challenge, expression of MHC class I and II antigens on interstitial CD45^pos^ CD11b^pos^ SiglecF^neg^ lung cells was only marginally increased in control mice, while the expression stayed high in mice with chronic schistosomiasis (Fig. 3B).

The protective effect of bilharzia on subsequent viral challenge was not found in mice that were infected 12 weeks prior with single-sex cercariae (Fig. S8), which indicates that the eggs - rather than the worm itself - induce protection.

### Histopathology in mice infected with PVM

We scored the extent of histopathology of the bronchial mucosae and of the lung parenchyma before and after challenge with PVM (Fig. 3A). The combined score is shown in Fig. 3C (scores for the individual parameters are shown in Fig. S5, B–F). Early in the infection with PVM (day 0 (Fig. 2D) and day 4 (Fig. 3A and S5B)), mice with chronic schistosomiasis had marked goblet cell hyperplasia, which disappeared later on. At day 4 and 7 after viral challenge, the combined score was significantly lower in *Schistosoma*-infected mice when compared to controls.

### Inflammatory processes with a strong Th2 component can protect from viral attack

**Figure 8:**
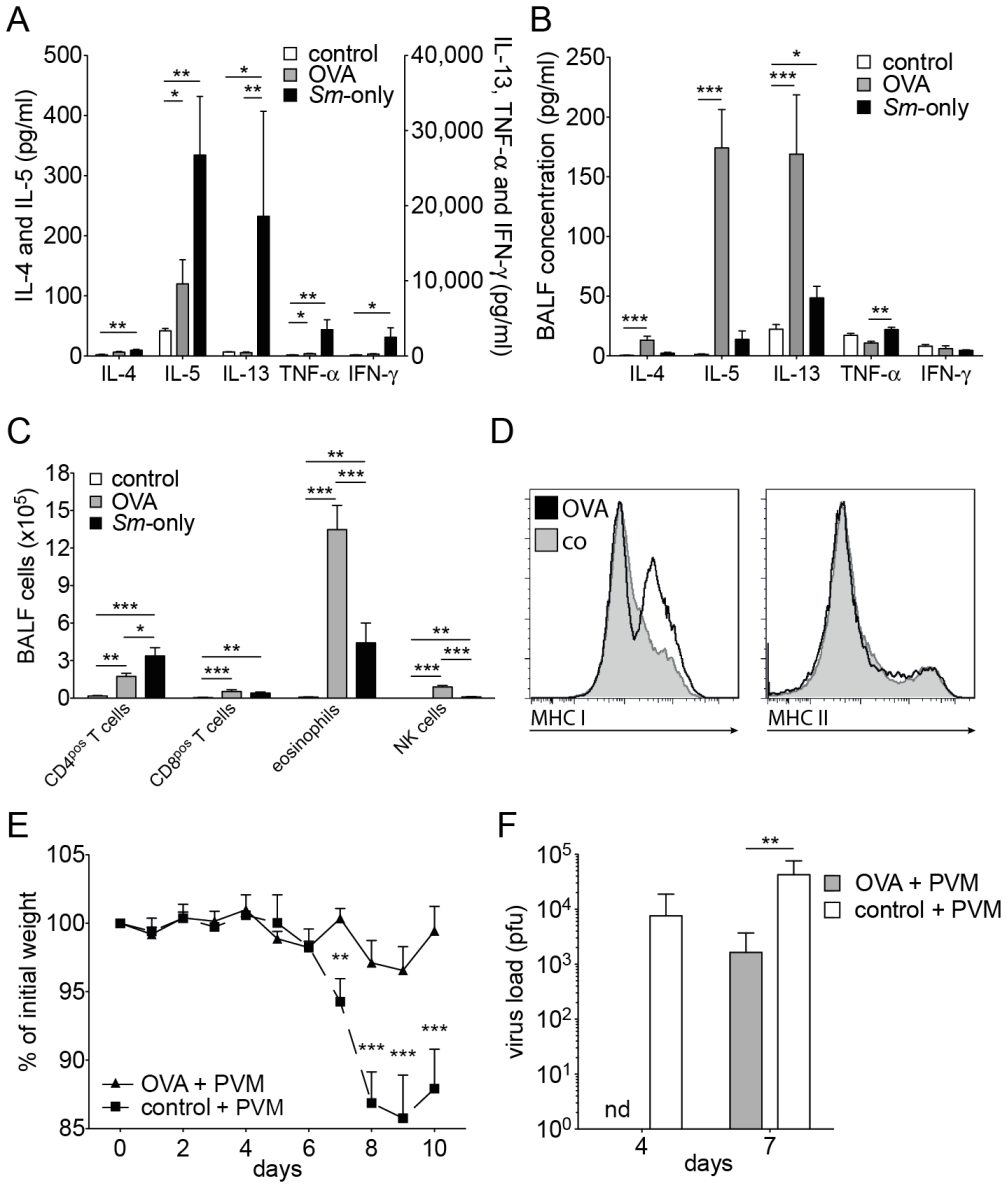
Resistance of C57BL/6 mice with inflammatory airway processes to challenge with PVM. **A**, **B**, Cytokine levels in the serum (A) and BALF (B) 24 h after the last of 3 consecutive days of aerosolised OVA of OVA-immunised mice (C57BL/6J) compared to mice infected with *S. mansoni* and control mice. The data shown are pooled from 4–8 mice in 3 individual experiments; **C**, Number of cells of various cell types in the BALF of mice 24 h after the last of 3 consecutive days of treatment with aerosolised OVA of OVA-immunised mice (C57BL/6J) compared to mice infected with *S. mansoni* and control mice. The data are from 10–14 mice in 3–5 individual (depending on the time point) experiments; **D**, FACS profiles of the expression of MHC class I and II antigens on interstitial CD45^pos^ CD11b^pos^ SiglecF^neg^ lung cells of C57BL/6J mice 24 h after the last of 3 consecutive days of aerosolised OVA challenge of OVA-immunised mice (thick lines) and control mice (shaded grey). Data shown is representative for 6 mice from 2 experiments; **E**, Relative weight loss of OVA-immunised mice (C57BL/6J) challenged with a sublethal dose (200 pfu/mouse) of PVM 24 h after the last of 3 consecutive days of aerosolised OVA and control mice at the indicated times after challenge. The data are cumulative from 10–16 mice in 3 individual experiments. The controls were obtained separately and are the same as in Fig. 7A. **F**, Viral load, expressed as pfu, in the right lobes of the lungs of mice, treated as described in Fig. 8E, at day 4 and 7 after challenge with PVM. The controls were obtained separately and are the same as in Fig. 7E. The data from OVA-immunised mice are derived from 2 and 3 mice (day 4 and 7, respectively). Error bars represent SEM. nd: not detectable. Significant differences are marked: *, *p*≤0.05; **, *p*≤0.01; ***, *p*≤0.001.

Another factor that can lead to increased Muc5ac production is an increased level of IL-13 in the lungs [38], as is found in pulmonary inflammatory processes, in particular in those with a strong Th2 component. Indeed, in a model of allergic airway inflammation [25] we saw type II inflammatory responses similar to those in the lungs of mice with schistosomiasis, but more pronounced. Levels of IL-5 in serum were high (Fig. 8A). In BALF high levels of IL-4, IL-5 and IL-13 were found (Fig. 8B). We found increased numbers of CD4^pos^ and CD8^pos^ T cells, NK cells, and eosinophils in BALF (Fig. 8C). The expression of MHC class I, but not of MHC class II antigens on CD45^pos^ CD11b^pos^ SiglecF^neg^ interstitial lung cells was increased (Fig. 8D). When challenged with 200 pfu/mouse PVM, ovalbumin-immunised and -challenged mice were protected from loss of weight and signs of disease (Fig. 8E). At day 7 after viral challenge these mice had significantly lower viral titres than the control mice (Fig. 8F).

## Discussion

In this work we studied the immunological influence of murine schistosomiasis on a subsequent challenge with a respiratory virus. Mice that are chronically infected with an human Omani isolate of *S. mansoni* are relatively, but significantly protected from a viral respiratory challenge with low doses of influenza A virus or pneumonia virus of mice (PVM), and they survived challenges with normally lethal doses. The protection is caused by TNF-*α*-mediated goblet cell hyperplasia and concomitant Muc5ac production in the lungs of *S. mansoni*-infected mice.

### Th1- besides Th2- and Th17-mediated immunity in murine schistosomiasis

*S. mansoni* is thought to induce primarily Th2 immune responses in the chronic, egg-depositing stage of the disease [2], [10]. Indeed, *Schistosoma* eggs themselves are considered important drivers of Th2 responses, through principles secreted by them: IPSE-alpha-1 [49] and omega-1 [50], [51]. Th2 responses are thought to inhibit Th1 responses, either by direct inhibition or by a relative deficiency for the appropriate inducing cytokine. A careful analysis of the systemic (serum) cytokine levels in mice chronically infected with the Omani isolate revealed that type 1 (IFN-*γ*, TNF-*α*, GM-CSF), type 2 (IL-5, IL-13, to a lesser degree IL-4), and type 17 (IL-17) cytokines coexisted throughout the infection. The cytokines rose to high levels from week 8 of infection onwards. When probed for potency, CD4^pos^ and CD8^pos^ T cells from the spleen and mesenteric lymph nodes produced high levels of IFN-*γ* and TNF-*α*, besides IL-4, IL-13 and IL-17. The increase in production of Th2 cytokines was percentually higher than those of Th1 cytokines. IL-4 and IL-13 are indispensible in the gut to initiate tissue repair mechanisms [11] and to expel worm eggs [52], and IL-17 is necessary for protection from bacterial attack through the battered gut wall [53], [54]. TNF-*α*, IL-13 and IL-17 can all induce mucin production, both in the intestines and in the airways. Mucins play an important role in worm expulsion, Muc5ac can expell and harm certain nematodes [55]. Remarkably, for intestinal goblet cell hyperplasia in schistosomiasis signalling by IL-4/IL-13 is dispensible [56].

### Thelper cell polarisation

The notion that Th1 or Th2 polarisation occurs in a systemic fashion was strongly influenced by early studies on leishmaniasis. These showed an exclusive responsiveness and a different immunity, depending on the strain of mice (C57BL/6 and BALB/c) [57]. We compared C57BL/6 and BALB/c mice in their systemic response to *S. mansoni* and could not find fundamental differences. Can we explain previous, discordant results of other groups? First, some reports are compatible with our results [58], and in a meta-analysis, Yu et al. [59] found significantly increased TNF-*α* and IL-4 levels in human patients with schistosomiasis. Second, we did not infect with the commonly used Puerto Rican strain of *S. mansoni*. However, the Omani strain caused a disease that resembled the moderate splenomegaly syndrome (MSS) of mice [30], with very few eggs being dispersed to the lungs. Third, in some studies mice were treated with antibiotics, which are known to significantly affect the microbiome and therewith the immune response [17], [18], [60]. We did not find evidence for a systemic bacterial spread or LPS in the blood of infected mice [61]. Fourth, in many experiments, mice were not followed beyond week 8, only 2 weeks after the onset of oviposition Fifth, the majority of conclusions relied on experiments in which *ex vivo* production of cytokines by polyclonally stimulated T cells was measured [3], [62]. In this approach the potential activity rather than actual activity of regional T cells is gauged [63]. We strongly support the idea, that regional immune cells can exert exclusive regional functions, for instance the production of Th2 cytokines in the gut. These facilitate egg transition through the gut and limit inflammation in the gut [60]. Finally, the concept of mutually exclusive responses slowly becomes cracks: recent studies point towards a more plastic view on the response [64], [65], and cell fate seems to be fixed rather early in the differentiation pathway of a T cell. In this concept, specifically responsive cells can be called upon later, when demand is present [66], [67]. Early systemic Th1 responses in *S. mansoni* could function as a stabilisator of the enhancer “landscape”, allowing continuous access to a Th1-prone cell pool [68]. Indeed, several groups found evidence for dual or hybrid types of helper T cells [69]–[71]. We therefore argue that our model of murine schistosomiasis resembles early infection in human, in which, like in our model, Th1, Th2 and Th17 cells coexist.

### The role of TNF-*α* in schistosomiasis

High levels of TNF-*α* and IFN-*γ* are often implied in the pathogenesis of a severe form of human schistosomiasis, the hepato-splenic form [72], which could resemble Henderson's hypersplenomegaly syndrome (HSS). As outlined, we do not find evidence for HSS in our model. Also in the mild form, hepatic involvement in mice is more pronounced than in benign cases of human schistosomiasis. Also in MSS increased levels of TNF-*α* are found in liver tissue [73]. In mice both protective and deleterious effects have been described for TNF-*α*, together with evidence that granuloma-induced TNF production is necessary for the maturation of the adult worms [74], [75].

### Pulmonary milieu in murine schistosomiasis

In chronic murine schistosomiasis the lungs show low-grade inflammatory processes, with goblet cell hyperplasia and increased production of the gel-forming mucin Muc5ac (see Fig. S9 for a graphical summary of our data). Early in the infection Th1 cytokines were found in BALF, they stayed high with the exception of IFN-*γ*. Th2 cytokines also rose early, with the exception of IL-4. Pathology in our model was less pronounced than in that described by Crosby et al. [58], which could well be explained by the lower number of parasite eggs found in the lungs of our mice. The sustained high levels of IL-13 and IL-5 without a joined increase of IL-4 suggests that they were not derived from Th2-type helper cells, but rather from ILC2-type cells [76], [77]. However, we saw a relative decrease in number of ILCs during the course of schistosomiasis, likely arguing against an activated state of the ILCs [37]. Instead, our results indicate that sustained high levels of systemic TNF-*α* were primarily responsible for the goblet cell hyperplasia.

### Coinfection studies with *S. mansoni* and influenza virus or PVM

In this *Schistosoma*-moulded milieu respiratory viruses do not spread as easily as in a healthy environment. PMV did not expand as rapidly and was cleared with a lower expenditure of CD8^pos^ cytotoxic T cells, and therefore with less airway inflammation and involvement. As a consequence, a lower combined histopathology score was found. The virulent influenza virus strain hvPR8 is lethal to normal (Mx-competent) mice, unless they are pretreated with type-I or type III interferons, which induce the indispensible Mx protein. Mice with chronic schistosomiasis were to a certain extent resistant to strain hvPR8, indicating that the infection with *S. mansoni* had either induced the production of type I or type III interferons or that other protective mechanisms, like the production of Muc5ac [44], were active. We were unable to detect significant amounts of IFN-*α* by ELISA in mice with schistosomiasis, and reporter mice for IFN-*β* did not show any significant involvement of IFN-*β*. Therefore, we conclude that type I interferons did not play a significant role in the observed protection, although we cannot completely exclude the involvement of type III interferons [78]. High levels of isolated production of Muc5ac in the lung, as found in Muc5ac-transgenic mice, protects from infection with influenza A virus [44]. In these studies Muc5ac production was increased by a factor of 20, in our studies, mice with chronic schistosomiasis had a ten-fold induction of Muc5ac. Treatment of the mice with Etanercept revealed that upstream TNF-*α* was responsible for this induction. Indeed, Mx1-deficient mice with chronic schistosomiasis were relatively, but not absolutely protected from an infection with influenza A virus, and the protection was lost after treatment with Etanercept.

TNF-*α* does not seem to affect viral replication in primary infections [47], [79], [80]. However, TNF has a significant effect on immunopathology: it negatively regulates the extent of pathology [79], or increases pathology [80], depending on virus strain and study design. Therefore we favour the notion that was put forward by Ehre et al. [44]: mucus alone is sufficient to sequester virus and reduce viral entry into the lung cells. Less cytotoxic T cells were needed for viral control, which resulted in reduced tissue damage.

### The role of TNF-*α* in Muc5ac expression and goblet cell hyperplasia

TNF-*α* has a direct influence on Muc5ac transcription via the NF-κB pathway, and it induces expression of the EGFR [40]. NF-κB further modulates IL-13R*α*1-signalling [81]. Both the IL-13R*α*1 and the EGFR are essential in the development of goblet cell hyperplasia. It is debated, whether these factors work in parallel or whether engagement of the EGFR is making the goblet cell receptive for consecutive IL-13 signalling [82]. We saw that short-time treatment of mice with Etanercept, i.e. neutralising TNF-*α* in the continuing presence of IL-13, inhibited Muc5ac expression and interfered with protection to viral challenge. This suggests a role for TNF-*α* that is independent of IL-13. Indeed, it was clearly shown that the mere transgenic expression of Muc5ac, without the involvement of (IL-13-driven) inflammatory responses, is protective in influenza infections [44].

### Factors influencing protection

The increased resistance to viral attack and the increased immunological “alertness” in the lungs of mice with schistosomiasis is not dependent on worm passage through the lungs, because infections with single-sex cercariae did not protect from viral attack. Protection is reliably found after week eight of infection, thus after the onset of oviposition. This coincides with the first deposition of eggs in the liver and the subsequent granulomatous reaction, but more importantly, also the passage of the eggs through the intestinal wall. This causes considerable damage, leads to inflammation, the secretion of high amounts of IL-17, and a noticeable change in the stool constitution with first some, later more - and also bloody - mucoid stool. Conditions in the gut of mice with schistosomiasis are therefore very different from normal. Already under normal conditions, bacterial products can enter the body, enhance systemic innate immunity [36], and exert considerable influence also in distant compartments like the lung. Effective immunity to influenza virus is dependent on the existing microbiome [17], [18], with bacterial products of gram-positive bacteria and certain TLR-ligands (TLR2,-3,-4 or -9) as active principle, even when rectally applied [17]. Abt *et al*. showed that interferon-induced genes were responsible for this kind of protection [18]. We could show, that systemic IFN-*γ* responses were absent in TLR2,4-double-deficient mice with schistosomiasis. In IL-12R*β*2-deficient mice both IFN-*γ* and TNF-*α*were absent, pointing to an IL-12/IFN-*γ* axis in protection in mice with schistosomiasis.

### Allergic inflammation and viral infections

We then asked whether pulmonary inflammatory processes in general could lead to protection. We found that in a mouse model of asthma mice were protected from viral attack by PVM, to a similar degree as found in mice with schistosomiasis. Indeed, conditions in the lung were to a large degree comparable, with a strong Th2 component in the inflammation. These results were not unexpected, Th2 cytokines, in particular IL-13, are potent inducers of goblet cell hyperplasia and Muc5ac production and thus protective in viral attacks. Remarkably, in the recent pandemic influenza (H1N1) infection, asthma was independently associated with a lower risk of dying during hospitalisation [83]. We interpret these results as a reflection of an increased immune alertness, with goblet cell hyperplasia and Muc5ac production. This response will initially protect the host from pneumotropic viruses, but in chronic conditions it may be detrimental for the host because of ensuing, debilitating chronic inflammatory processes like COPD [84].

### Other helminths will exert other effects

We do not think that our findings can be transferred to all helminth infections. In infections in which the parasite deploys systemic immunosuppression, as does for instance *Heligmosomoides polygyrus* [85], [86], or induces only a weak Th1 response, the outcome can be different. Indeed, two recent articles supported this notion. In a murine model, infection with *H. polygyrus* and *S. mansoni* eggs (a complete infectious cycle was not tested) could reactivate the mouse *γ*-herpes virus MHV68 *in vivo*, via an IL-4- and Stat6-dependent pathway[87]. In a second study, *Trichinella spirali*s and *H polygyrus* both could inhibit specific T-cell responses to murine norovirus, or to influenza virus (only tested with *T. spiralis*). Immune modulation was shown to be dependent on a Stat6-dependent alternative activation of macrophages and their secreted product Ym1 [88]. In both cases, Th2-type immune responses are induced, without previous Th1-type immunity. Unfortunately, no data were provided for the lung environment.

In schistosomiasis, we did not find evidence for a generalised immune suppression. The similarity of *S. mansoni*-induced and allergen-induced pulmonary inflammation rather predicts a more severe course, when allergy and schistosomiasis coexist. The immune regulatory activity of T regulatory cells and IL-10 is therefore most likely secondary to inflammation and antigen-specific [54]. Remarkably, in schistosomiasis, a disease with at least 200 million people a year needing treatment, little tenable information is available regarding comorbidities. Of course, many variables can influence the outcome of coinfections, as discussed extensively by Abruzzi and Fried [6].

In conclusion, our results shed a new light both on the biology of schistosomiasis and the immune response to the parasite. Contrary to previous reports, we do not find the immune responses to be polarised systemically, but they reflect local need for defence. From an evolutionary point of view, the parasite would initially benefit from an increased barrier function towards common pathogens like respiratory tract viruses.

## Supporting Information

Figure S1 **Determination of the optimal cercarial dose of** ***S. mansoni*** **that leads to a chronic disease. A**, five C57BL/6J mice were exposed to different number of cercariae and analysed 12 weeks after infection for worm burden (Δ), worm pairs (**⧫**), infection rate (**○**) and weight of liver (**▪**) and spleen (**□**). **B**, total number of eggs/liver 12 weeks after infection, relative to the cercarial dose. Data points represent mean data from 3–7 mice in one experiment. Error bars represent SEM. Grey zones indicate the optimal cercarial dose we have used in further experiments to obtain a chronic course of the disease.
(TIF)

Figure S2 **Serum cytokine levels in BALB/cJ mice at indicated time points after infection with** ***S. mansoni*** **. A**, signature Th1 cytokines IFN-*γ* and TNF-*α* and **B, D**, signature Th2 cytokines IL-5, IL-13 and IL-4 were measured, as well as **C, D**, IL-6, IL-17 and IL-2. The data points represent means from 2–7 mice. Error bars represent SEM. Significant differences between each time point and control mice (day 0) are marked: *, *p*≤0.05; **, *p*≤0.01; ***, *p*≤0.001.
(TIF)

Figure S3 **Serum IFN-*γ*(A) and TNF-*α*(B) levels in C57BL/6J (black line), TLR2^–/–^/4^–/–^ (green line) and IL-12R*β*2^–/–^ (blue dotted line) mice at indicated time points after infection with** ***S. mansoni*** **.** Error bars represent SEM. Significant differences between each time point and control mice (day 0) are marked: *, *p*≤0.05; **, *p*≤0.01; ***, *p*≤0.001.
(TIF)

Figure S4 **Serum endotoxin levels in C57BL/6 mice at indicated time points after infection with** ***S. mansoni*** **.** Serum endotoxin levels were determined by LAL assay. The data points represent means from 11, 4 and 12 mice at week 0, 5, and 12, respectively. Error bars represent SEM. ANOVA analysis (Kruskall-Wallis): p = 0.0488.
(TIF)

Figure S5 **Histo-pathological scores of the lungs of C57BL/6J mice with chronic schistosomiasis. A**, basal values at 12 weeks after infection with *S. mansoni*; **B–F**, scores of mice with schistosomiasis (12 wks; black) and control animals (white) at indicated times after a sublethal dose (200 pfu/mouse) of PVM. Columns represent mean results from 2–3 mice per group. Error bars represent SEM. Scoring: 0: normal; 1: minor; 2: intermediate; 3: markedly increased. Absence of bars represents normal (0) scoring.
(TIF)

Figure S6 **Analysis of ILCs in the lungs of control mice and at indicated time points after infection with** ***S. mansoni*** **.** ILCs were prepared from the right lobes of the lungs. **A**, gating strategy. Lin: Lineage markers. The plots are representative for n = 10 (control), 4 (week 2), 2 (week 5), 4 (week 9) and 10 (week 12) mice. **B**, total number of cells that are Lin-negative and positive for the markers CD25, CD90 and T1-ST2. Significant differences between groups are marked: *, *p*≤0.05; **, *p*≤0.01; ***, *p*≤0.001.
(TIF)

Figure S7 **Type I interferon levels in mice infected with** ***S. mansoni*** **at week 12 of infection. A**, IFN-*α* levels in BALF; **B**, IFN-*β* levels in indicated organs (RLU: relative luciferase units) Error bars represent SEM. Significant differences between each time point and control mice (day 0) are marked: *, *p*≤0.05; ***, *p*≤0.001.
(TIF)

Figure S8 **Coinfection experiments with** ***S. mansoni*** **-infected mice and pneumonia virus of mice (PVM).** Mice were i.n. challenged at day 0 with 200 pfu of virus. Relative weight loss of C57BL/6J mice infected with 100 cercariae of a single sex (cercariae obtained from snails infected with only 1 miracidium) (12 wks) and control animals at indicated times. Data are pooled from 13 mice in 2 experiments. Error bars represent SEM. Significant differences between data points from normal and single sex infection with *S. mansoni* are marked: *, *p*≤0.05; **, *p*≤0.01.
(TIF)

Figure S9 **Graphical summary.** Representation of pulmonary conditions. Upper left: normal condition. Upper right: chronic schistosomiasis. Lower left: infection with a respiratory virus. Lower right: secondary infection with a respiratory virus in chronic schistosomiasis.
(TIF)

Table S1 ***Ex vivo*** **stimulation of cells.** Pulmonary lymph nodes (pLN), spleens and mesenteric lymph nodes (mLN) were taken from mice on the C57BL/6J background and single cell suspensions were prepared. At week 0 not enough cells could be prepared from the pLN to perform *in vitro* stimulation. Cells were then incubated for 4 h in complete medium containing phorbol 12-myristate 13-acetate (PMA, 20 ng/ml), Ionomycin (500 ng/ml) and Brefeldin A (1 µg/ml). Cells were stained extracellularly and, after fixation, stained intracellularly with fluorochrome-labelled anti-TNF-*α* (MP6-XT22), anti-IFN-*γ* (XMG1.2), anti-IL-4 (11B11) and anti IL-13 (eBio13A), using the IntraSure fixation and permeabilisation kit (all from BD Biosciences, except for anti IL-13, which was from eBioscience). The percentage of cells positive for the indicated cytokine is given.
(TIF)
