# Supplementary figures and images for: S. mansoni Bolsters Anti-Viral Immunity in the Murine Respiratory Tract
Source: PLoS One. 2014 Nov 14;9(11):e112469. doi: 10.1371/journal.pone.0112469 (PMC4232382; doi:10.1371/journal.pone.0112469)

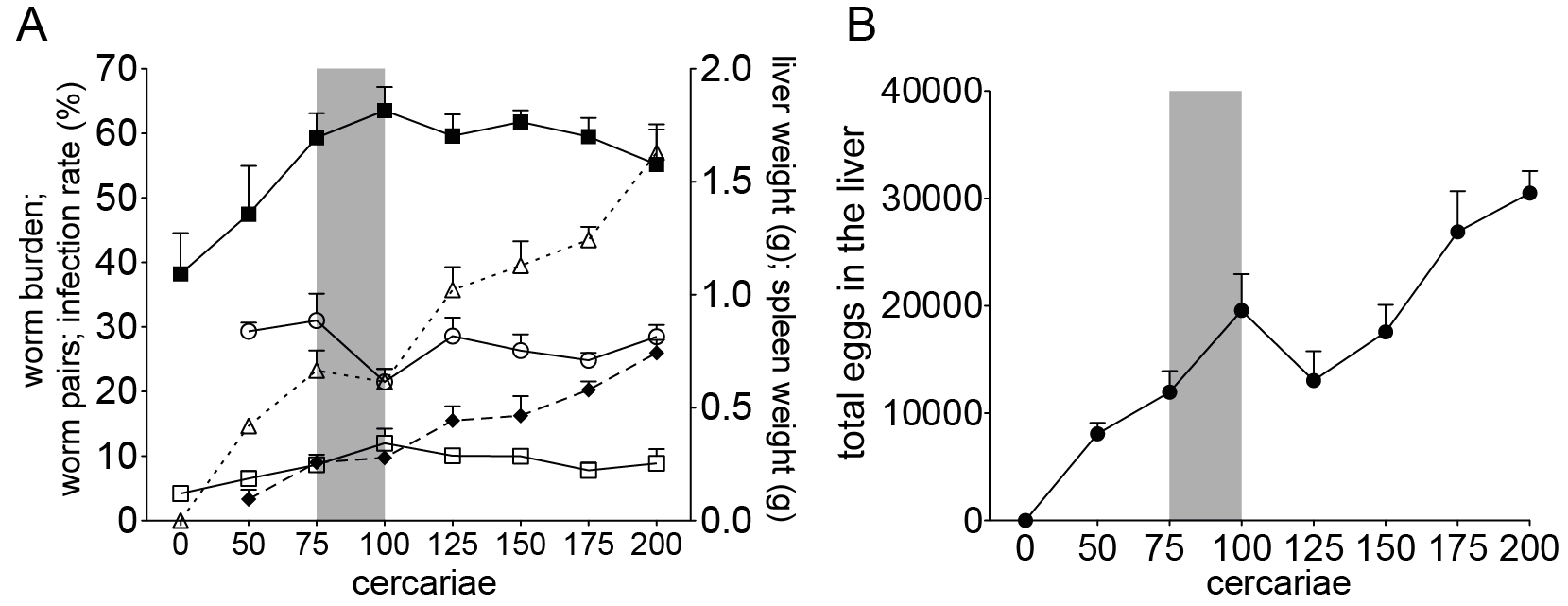

Supplement: Figure S1 — Determination of the optimal cercarial dose of S. mansoni that leads to a chronic disease. A, five C57BL/6J mice were exposed to different number of cercariae and analysed 12 weeks after infection for worm burden (Δ), worm pairs (⧫), infection rate (○) and weight of liver (▪) and spleen (□). B, total number of eggs/liver 12 weeks after infection, relative to the cercarial dose. Data points represent mean data from 3–7 mice in one experiment. Error bars represent SEM. Grey zones indicate the optimal cercarial dose we have used in further experiments to obtain a chronic course of the disease. (TIF) [file pone.0112469.s001.tif]

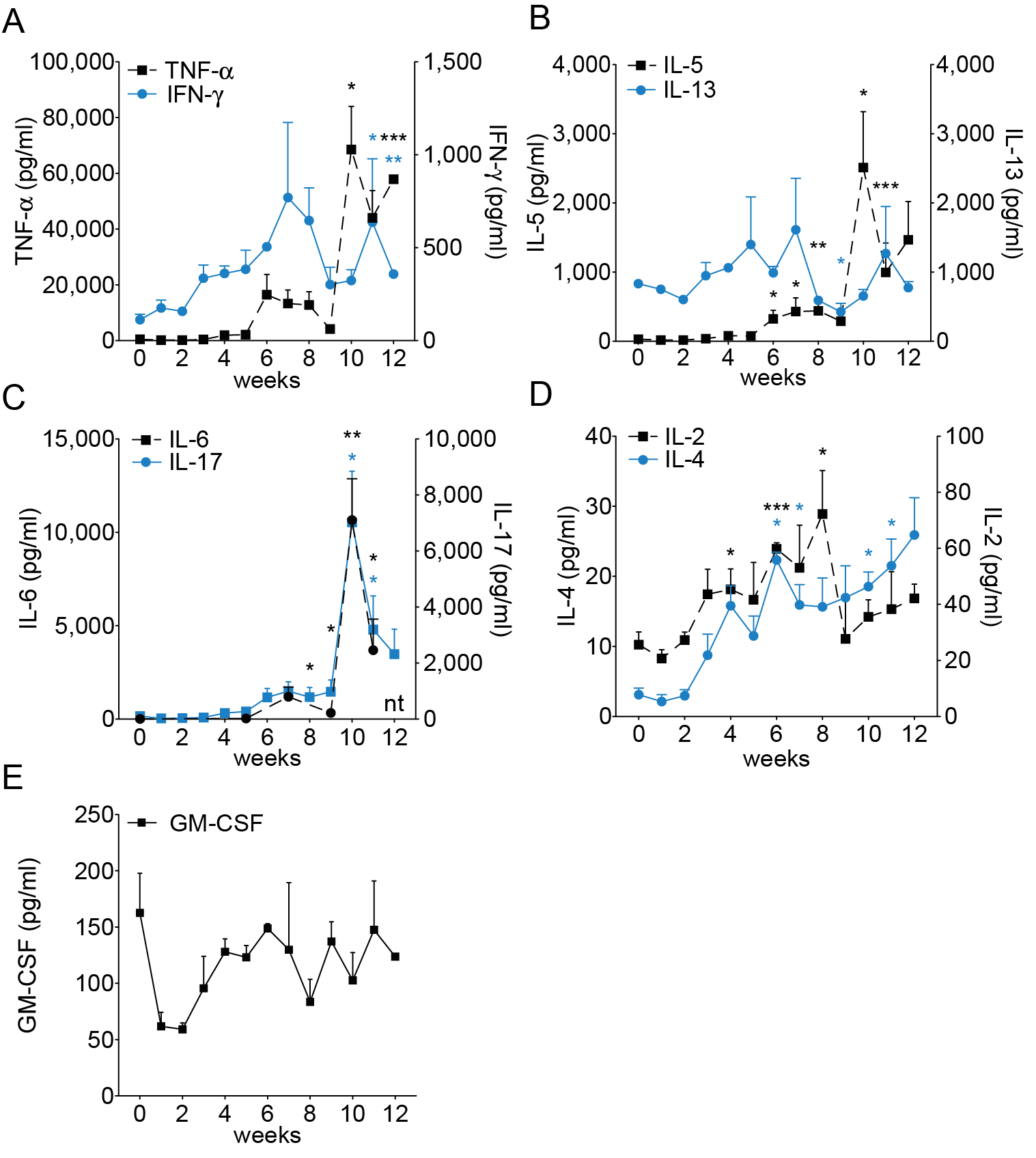

Supplement: Figure S2 — Serum cytokine levels in BALB/cJ mice at indicated time points after infection with S. mansoni . A, signature Th1 cytokines IFN-γ and TNF-α and B, D, signature Th2 cytokines IL-5, IL-13 and IL-4 were measured, as well as C, D, IL-6, IL-17 and IL-2. The data points represent means from 2–7 mice. Error bars represent SEM. Significant differences between each time point and control mice (day 0) are marked: *, p≤0.05; **, p≤0.01; ***, p≤0.001. (TIF) [file pone.0112469.s002.tif]

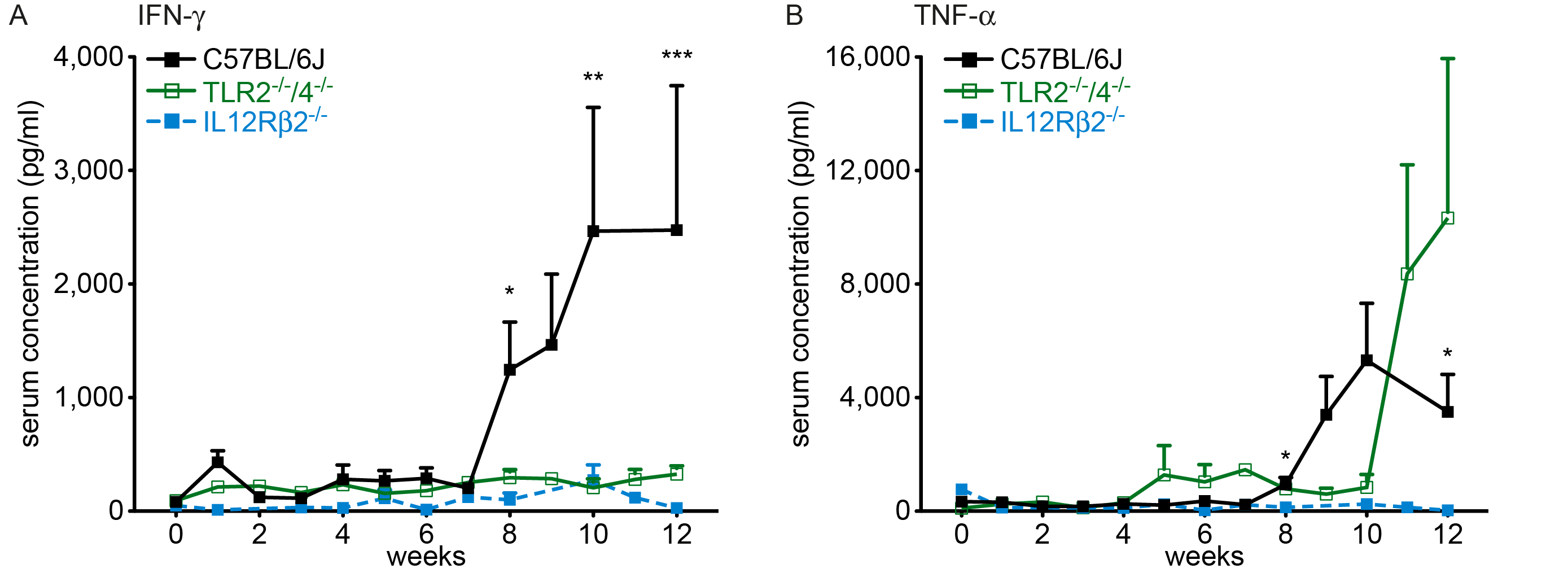

Supplement: Figure S3 — Serum IFN-γ(A) and TNF-α(B) levels in C57BL/6J (black line), TLR2–/–/4–/– (green line) and IL-12Rβ2–/– (blue dotted line) mice at indicated time points after infection with S. mansoni . Error bars represent SEM. Significant differences between each time point and control mice (day 0) are marked: *, p≤0.05; **, p≤0.01; ***, p≤0.001. (TIF) [file pone.0112469.s003.tif]

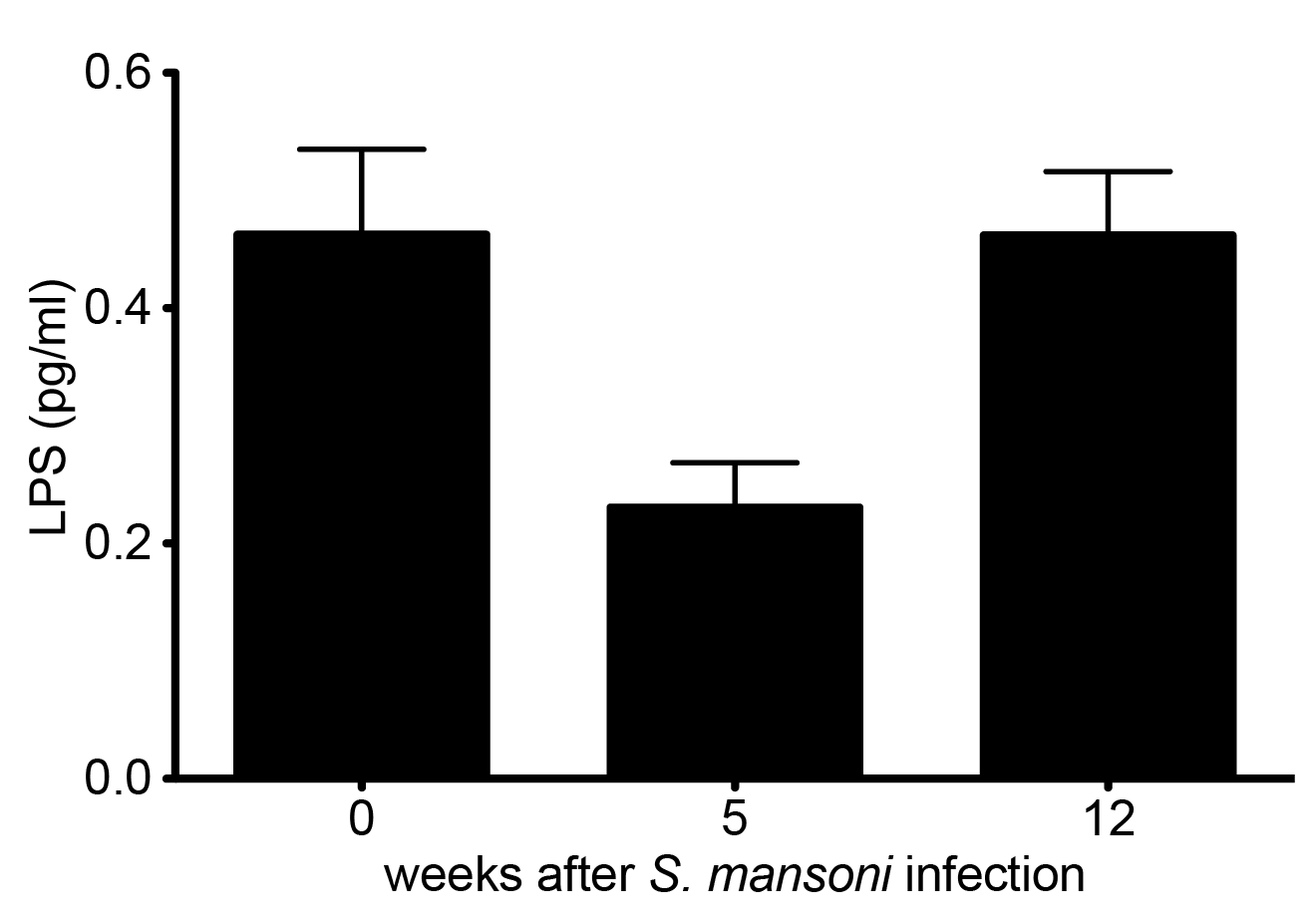

Supplement: Figure S4 — Serum endotoxin levels in C57BL/6 mice at indicated time points after infection with S. mansoni . Serum endotoxin levels were determined by LAL assay. The data points represent means from 11, 4 and 12 mice at week 0, 5, and 12, respectively. Error bars represent SEM. ANOVA analysis (Kruskall-Wallis): p = 0.0488. (TIF) [file pone.0112469.s004.tif]

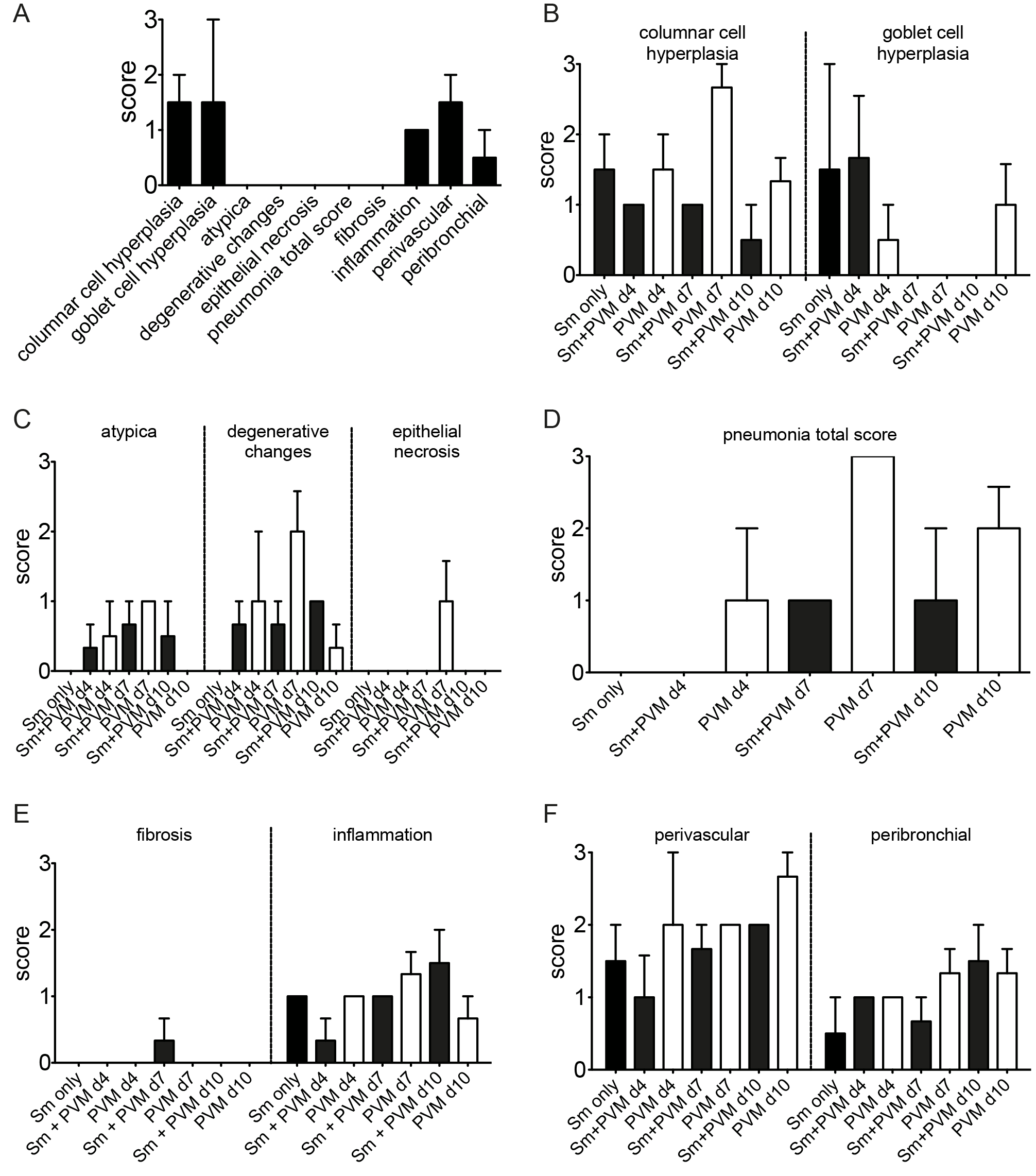

Supplement: Figure S5 — Histo-pathological scores of the lungs of C57BL/6J mice with chronic schistosomiasis. A, basal values at 12 weeks after infection with S. mansoni; B–F, scores of mice with schistosomiasis (12 wks; black) and control animals (white) at indicated times after a sublethal dose (200 pfu/mouse) of PVM. Columns represent mean results from 2–3 mice per group. Error bars represent SEM. Scoring: 0: normal; 1: minor; 2: intermediate; 3: markedly increased. Absence of bars represents normal (0) scoring. (TIF) [file pone.0112469.s005.tif]

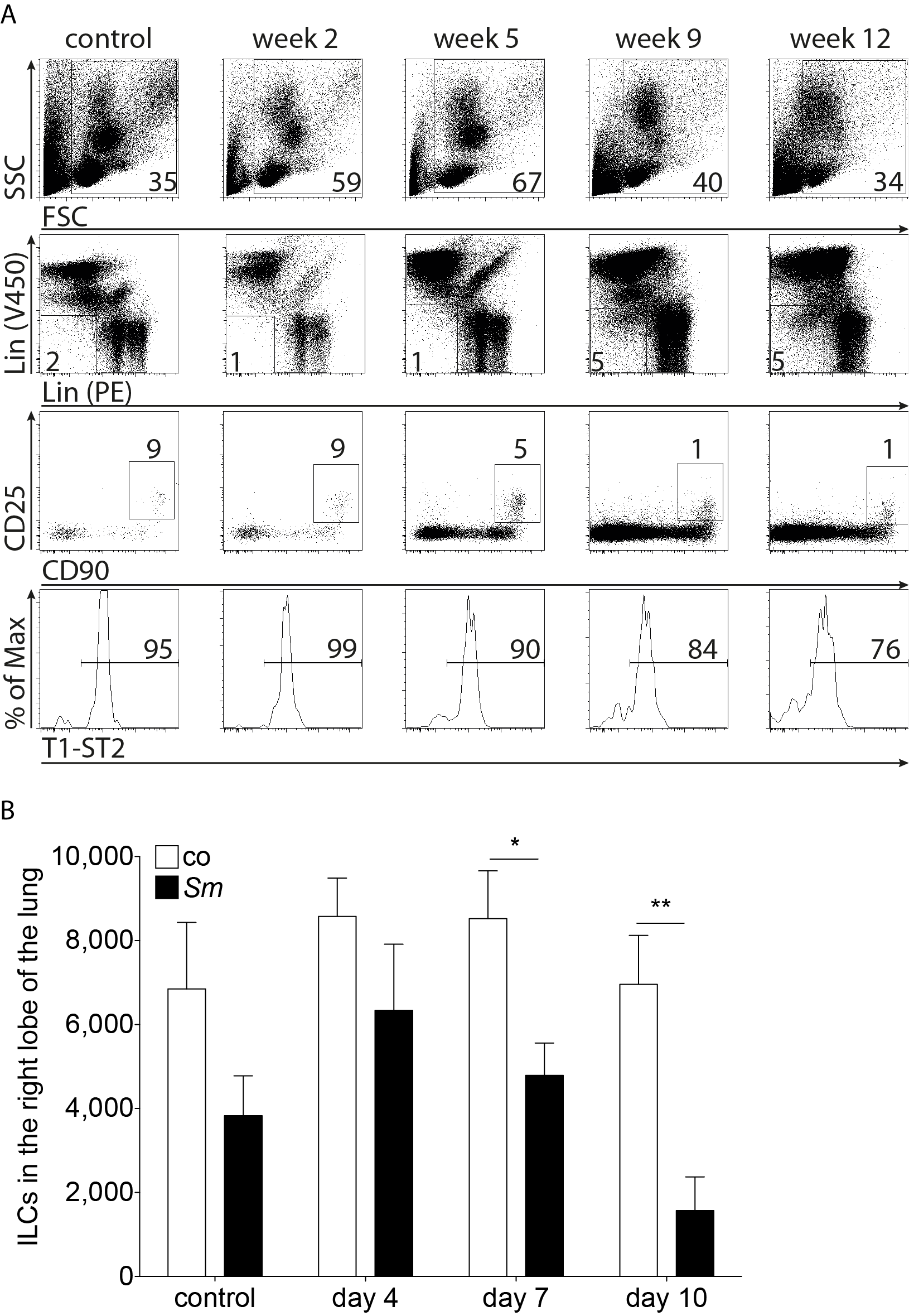

Supplement: Figure S6 — Analysis of ILCs in the lungs of control mice and at indicated time points after infection with S. mansoni . ILCs were prepared from the right lobes of the lungs. A, gating strategy. Lin: Lineage markers. The plots are representative for n = 10 (control), 4 (week 2), 2 (week 5), 4 (week 9) and 10 (week 12) mice. B, total number of cells that are Lin-negative and positive for the markers CD25, CD90 and T1-ST2. Significant differences between groups are marked: *, p≤0.05; **, p≤0.01; ***, p≤0.001. (TIF) [file pone.0112469.s006.tif]

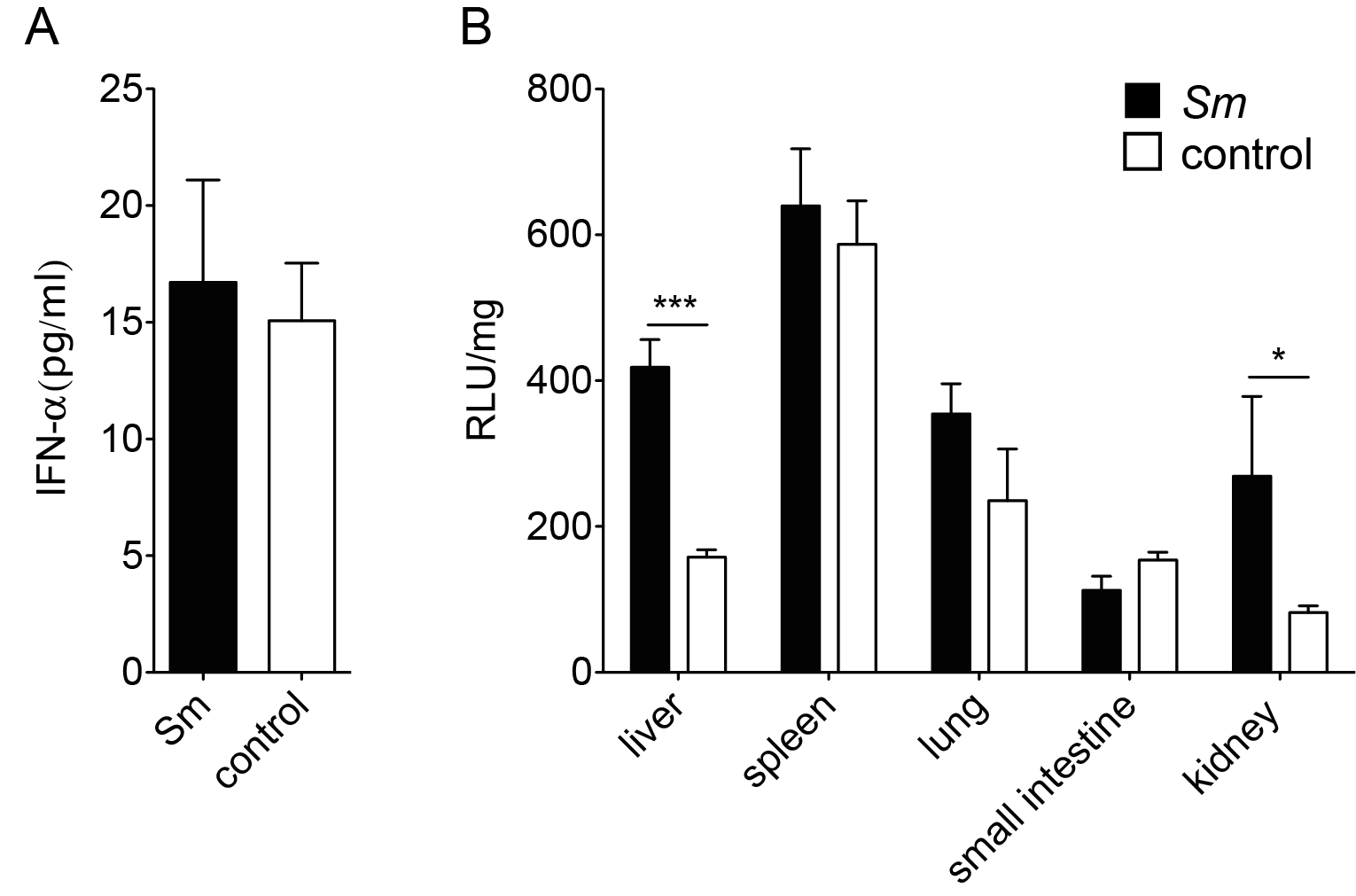

Supplement: Figure S7 — Type I interferon levels in mice infected with S. mansoni at week 12 of infection. A, IFN-α levels in BALF; B, IFN-β levels in indicated organs (RLU: relative luciferase units) Error bars represent SEM. Significant differences between each time point and control mice (day 0) are marked: *, p≤0.05; ***, p≤0.001. (TIF) [file pone.0112469.s007.tif]

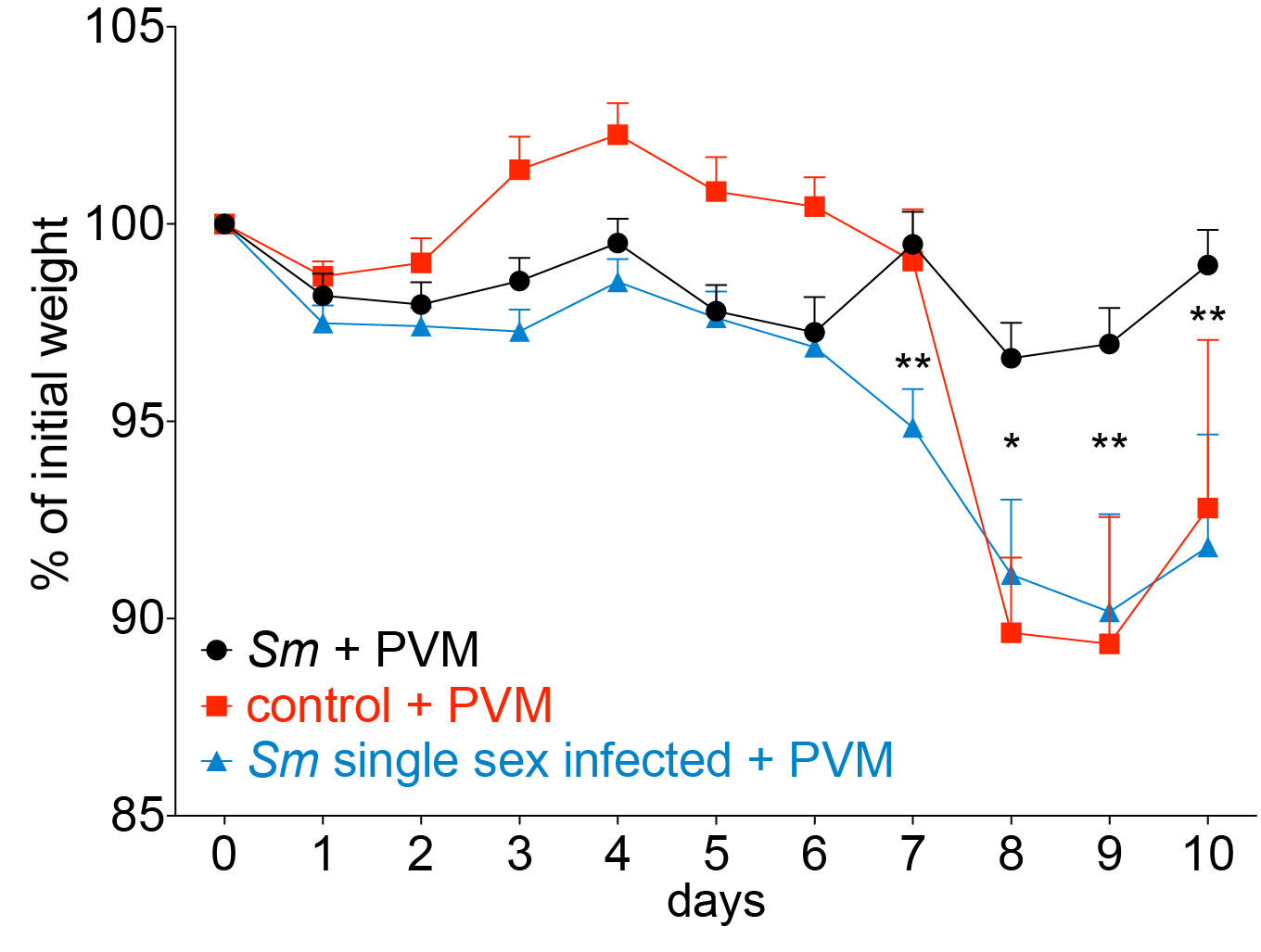

Supplement: Figure S8 — Coinfection experiments with S. mansoni -infected mice and pneumonia virus of mice (PVM). Mice were i.n. challenged at day 0 with 200 pfu of virus. Relative weight loss of C57BL/6J mice infected with 100 cercariae of a single sex (cercariae obtained from snails infected with only 1 miracidium) (12 wks) and control animals at indicated times. Data are pooled from 13 mice in 2 experiments. Error bars represent SEM. Significant differences between data points from normal and single sex infection with S. mansoni are marked: *, p≤0.05; **, p≤0.01. (TIF) [file pone.0112469.s008.tif]

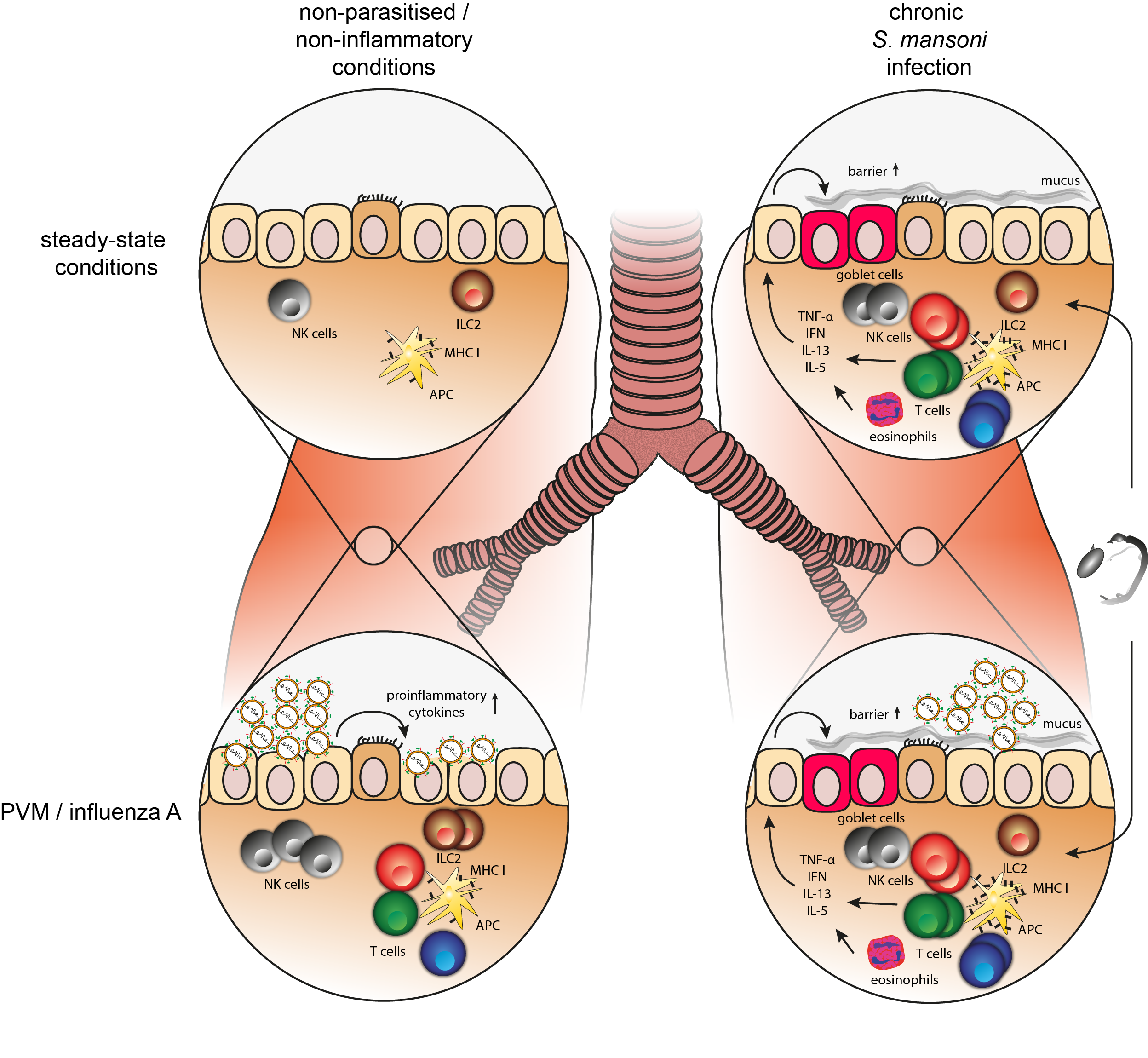

Supplement: Figure S9 — Graphical summary. Representation of pulmonary conditions. Upper left: normal condition. Upper right: chronic schistosomiasis. Lower left: infection with a respiratory virus. Lower right: secondary infection with a respiratory virus in chronic schistosomiasis. (TIF) [file pone.0112469.s009.tif]

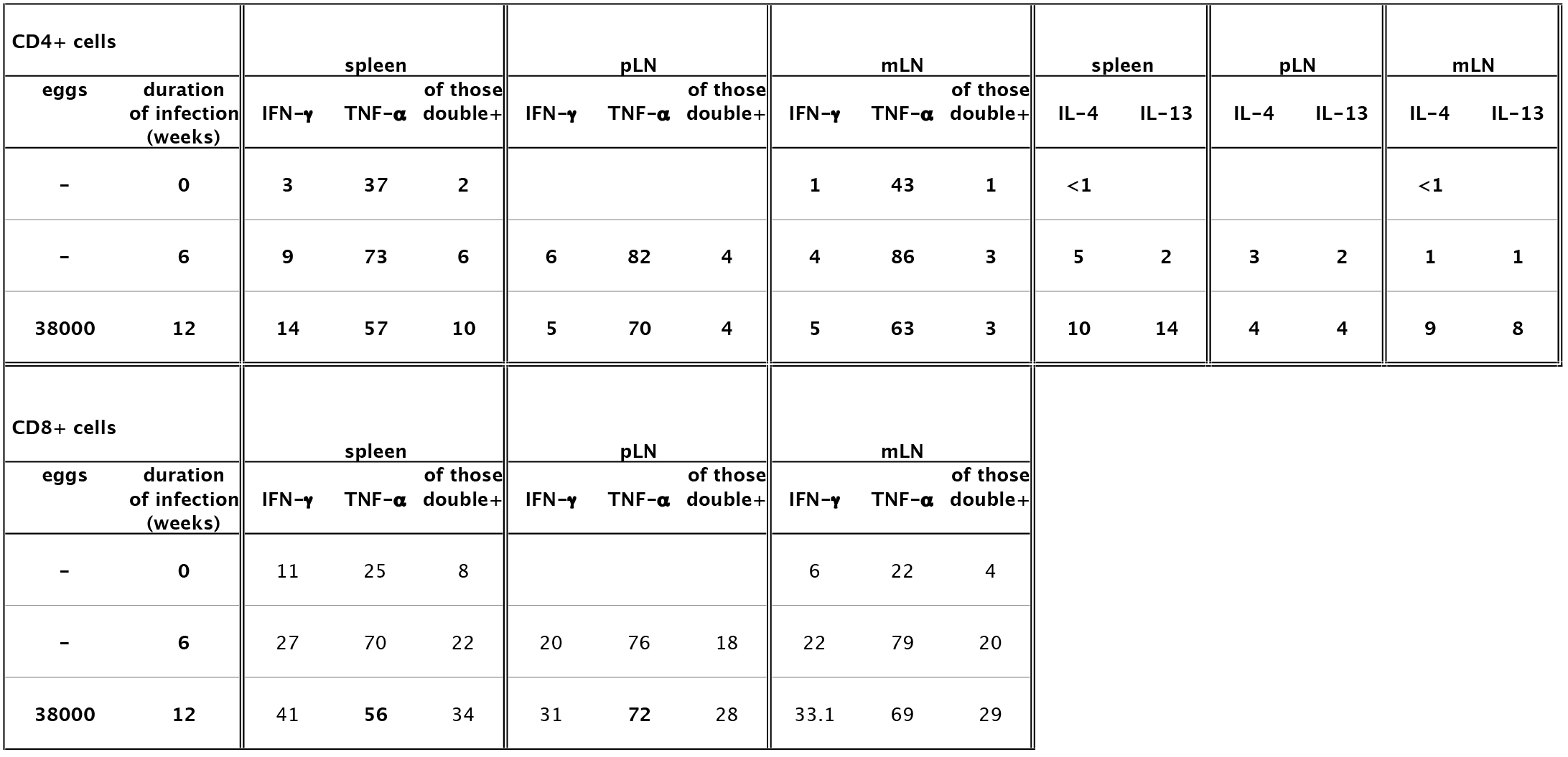

Supplement: Table S1 — Ex vivo stimulation of cells. Pulmonary lymph nodes (pLN), spleens and mesenteric lymph nodes (mLN) were taken from mice on the C57BL/6J background and single cell suspensions were prepared. At week 0 not enough cells could be prepared from the pLN to perform in vitro stimulation. Cells were then incubated for 4 h in complete medium containing phorbol 12-myristate 13-acetate (PMA, 20 ng/ml), Ionomycin (500 ng/ml) and Brefeldin A (1 µg/ml). Cells were stained extracellularly and, after fixation, stained intracellularly with fluorochrome-labelled anti-TNF-α (MP6-XT22), anti-IFN-γ (XMG1.2), anti-IL-4 (11B11) and anti IL-13 (eBio13A), using the IntraSure fixation and permeabilisation kit (all from BD Biosciences, except for anti IL-13, which was from eBioscience). The percentage of cells positive for the indicated cytokine is given. (TIF) [file pone.0112469.s010.tif]
